# An Iterative Genetic and Dynamical Modelling Approach Identifies Novel Features of the Gene Regulatory Network Underlying Melanocyte Development

**DOI:** 10.1371/journal.pgen.1002265

**Published:** 2011-09-01

**Authors:** Emma R. Greenhill, Andrea Rocco, Laura Vibert, Masataka Nikaido, Robert N. Kelsh

**Affiliations:** 1 Department of Biology and Biochemistry and Centre for Regenerative Medicine, University of Bath, Bath, United Kingdom; 2 Department of Mathematics, University of Bath, Bath, United Kingdom; University of Pennsylvania School of Medicine, United States of America

## Abstract

The mechanisms generating stably differentiated cell-types from multipotent precursors are key to understanding normal development and have implications for treatment of cancer and the therapeutic use of stem cells. Pigment cells are a major derivative of neural crest stem cells and a key model cell-type for our understanding of the genetics of cell differentiation. Several factors driving melanocyte fate specification have been identified, including the transcription factor and master regulator of melanocyte development, Mitf, and Wnt signalling and the multipotency and fate specification factor, Sox10, which drive *mitf* expression. While these factors together drive multipotent neural crest cells to become specified melanoblasts, the mechanisms stabilising melanocyte differentiation remain unclear. Furthermore, there is controversy over whether Sox10 has an ongoing role in melanocyte differentiation. Here we use zebrafish to explore in vivo the gene regulatory network (GRN) underlying melanocyte specification and differentiation. We use an iterative process of mathematical modelling and experimental observation to explore methodically the core melanocyte GRN we have defined. We show that Sox10 is not required for ongoing differentiation and expression is downregulated in differentiating cells, in response to Mitfa and Hdac1. Unexpectedly, we find that Sox10 represses Mitf-dependent expression of melanocyte differentiation genes. Our systems biology approach allowed us to predict two novel features of the melanocyte GRN, which we then validate experimentally. Specifically, we show that maintenance of *mitfa* expression is Mitfa-dependent, and identify Sox9b as providing an Mitfa-independent input to melanocyte differentiation. Our data supports our previous suggestion that Sox10 only functions transiently in regulation of *mitfa* and cannot be responsible for long-term maintenance of mitfa expression; indeed, Sox10 is likely to slow melanocyte differentiation in the zebrafish embryo. More generally, this novel approach to understanding melanocyte differentiation provides a basis for systematic modelling of differentiation in this and other cell-types.

## Author Summary

In a multicellular organism, one genome is used to make numerous different cell-types. This must require the activity of all these genes to be configured into multiple distinct and stable active states, each corresponding to one of the different cell-types characteristic of a tissue. The stable active states of differentiated cell-types contrast with the different, and transient, states characteristic of multipotent stem cells. We know little of the key features of these states that regulate the switch of a stem cell to stable differentiation. Here we examine this issue in the melanocyte, a genetically well-characterised cell-type, using a combination of dynamic mathematical modelling and experimental manipulation. In humans, disruption of the melanocyte state results in congenital and degenerative pigmentary diseases, whereas their destabilisation is likely to be an important factor in initiating melanoma. Our work predicts, validates, and identifies several novel features to the gene regulatory network of the zebrafish melanocyte, including one stabilising the differentiated state. Our study demonstrates the utility of this systems biology approach to understanding the genetic basis for differentiated cell states.

## Introduction

Understanding the mechanisms of generation of differentiated cell-types from multipotent precursors is a fundamental aspect of development, with profound implications for the therapeutic use of stem cells. Whilst numerous transcription factors mediating fate choice from stem cells have been characterised, we still lack a robust understanding of how these factors and their target differentiation genes interact to form the gene regulatory networks (GRNs) that result in stable differentiation. At the time of fate specification, a multipotent cell's GRN is configured so as to allow multiple fates to be chosen; after specification this GRN must shift to a new stable state to establish commitment to, and full differentiation of, a specific fate. *Tour de force* studies of the early development of the sea urchin embryo have become perhaps the most completely understood example [1]. These studies, amongst others, have identified two key themes of fate specification, that the adopted fate becomes stabilized by factors initiating positive feedback loops and that these then are reinforced by activation of repressors of alternative fates [2]. Increasingly it is becoming clear that mathematical modelling of these proposed networks is very informative for a rigorous understanding of their properties [3]–[5], but this remains rare, especially for vertebrate systems.

Vertebrate melanocytes (melanophores in fish, amphibians and reptiles) are critical for body pigmentation and play roles, for example, in mate recognition and protection against UV light. Numerous diseases result from failures of melanocyte specification (e.g. Waardenburg syndromes), differentiation (albinism), survival (vitiligo) or control of proliferation (melanoma) [6]. Melanocytes are genetically amongst the best characterised cell-types, with a long history of genetic analysis in mammals [7], but so far these data have not been used to generate mathematical models of melanocyte differentiation. Embryonic melanocytes are derived from the neural crest [8]–[10] and in the adult are renewed from dormant melanocyte stem cells [11]. Melanocyte specification centers on the transcriptional activation of Mitf, a bHLH-LZ transcription factor that is a master regulator of melanocyte differentiation [12], [13]. Key target genes of Mitf include those encoding the melanogenic enzymes Dopachrome tautomerase (Dct), Tyrosinase (Tyr) and Tyrosinase-related protein 1 (Tyrp1) and the melanosome structural protein Silver (Si). The Sox transcription factor Sox10 is also crucial for melanocyte development, where it contributes to melanocyte fate-specification by transcriptional activation of *Mitf*, consistent with the association of SOX10 with Waardenburg syndrome in humans [14]–[20].

Given that both MITF and SOX10 are frequently mutated in melanoma [21] and that MITF itself is considered to be a lineage addiction oncogene [22], understanding the melanocyte GRN is of crucial importance. However, controversy surrounds the precise role of Sox10, with in vivo data from zebrafish arguing that an ongoing role in melanocyte differentiation is not required in this organism [16], while in vitro data from mouse indicates that Sox10 may contribute to expression of melanocyte differentiation genes, *Dct* and *Tyr* [23]–[27]. We here combine experimental and mathematical modelling approaches to examine this issue in more detail in zebrafish.

We document the rapid loss of Sox10 from differentiating melanocytes in zebrafish embryos. We adapt a simple GRN model of sympathetic neuron development to the melanocyte case and assess its validity both experimentally and by mathematical modelling. This melanocyte model predicts that Sox10 *represses* expression of melanocyte differentiation genes, and that in this way Sox10 antagonizes Mitfa-mediated differentiation. Our analysis of gene expression patterns in zebrafish *sox10* and *mitfa* mutants provides strong support for this, and overexpression studies in zebrafish embryos confirm the repressive action of Sox10 on Mitfa-mediated transcription. The model also predicts that the turning off of *sox10* expression in differentiating melanocytes results from Mitfa-dependent repression of *sox10* transcription. We provide evidence that Mitfa can regulate *sox10* expression and that in vivo this effect is likely to be repressive and dependent upon Hdac1 function. We use simple mathematical modelling of this GRN, in conjunction with our previous experimental data, to establish that it is insufficient to explain stable melanocyte differentiation. We show that addition of further features, including a Sox10-independent positive feedback loop regulating *mitfa*, and a Sox10-independent weak activator of melanocyte differentiation gene expression, are sufficient to alter the GRN behaviour to allow stable differentiation of this cell-type and to explain our in vivo observations. Finally, we provide genetic evidence that Sox9b contributes to the second of these factors. The mathematical modelling of the melanocyte GRN proposed here provides the first such model for this important and well-characterised cell-type and provides the basis for future qualitative and quantitative refinement of our understanding of melanocyte differentiation. Our data supports the previous suggestion that Sox10 only functions transiently in *mitfa* expression and cannot be responsible for long-term maintenance of *mitfa* expression in zebrafish; indeed, Sox10 is likely to slow melanocyte differentiation in the embryo. This work has clear implications for the proposed model of sympathetic neuron differentiation, but also more broadly for our understanding of commitment to specific fates. Furthermore, these studies emphasize the importance of robust mathematical modelling of proposed GRNs to test their behaviour in a rigorous and quantitative manner.

## Results

### Sox10 expression is rapidly lost in differentiating melanocytes

**Figure 1:**
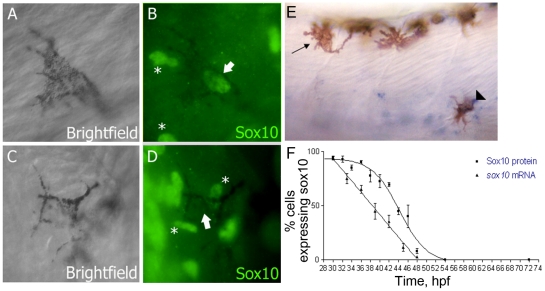
Sox10 is rapidly downregulated in differentiating melanocytes. A–D) Sox10 positive (A,B) and Sox10 negative (C,D) melanocytes from 33 hpf embryo are indicated by arrows. Non-pigmented cells expressing Sox10 are indicated (*). E) *sox10* in situ hybridisation on 33 hpf embryo showing both *sox10* positive (arrowhead) and *sox10* negative (arrow) melanocytes. F) Time-course of percentage of melanocytes showing Sox10 or *sox10* expression during melanocyte differentiation stages. Expression was examined in 20 pigmented cells from each of 5 fish (i.e. n = 100) at each time point.

Our previous studies have shown that *sox10* mRNA expression is rapidly lost from differentiating sensory neurons [28]. We asked whether this pattern was seen for *sox10* expression in differentiating melanocytes too. We used both whole-mount in situ hybridisation and immunofluorescence using a Sox10 antibody (kind gift of B. Appel) to evaluate the temporal persistence of Sox10 expression throughout a time-course (Figure 1). Melanocytes were selected at random from all dorso-ventral positions between the edge of the yolk and the end of the yolk sac extension. Expression was scored as the percentage of melanised cells showing detectable signal. The earliest signs of melanisation in trunk melanocytes in wild-type embryos are seen around 27 hpf [29]. At 30 hpf, almost all melanocytes showed detectable *sox10* and Sox10 expression, but this rapidly decreased, so that by c. 50 hpf, signal was not detected in any cells. This contrasts with the continuing expression of *mitfa* (data not shown and see Figure S3). We note that at this stage, melanocyte differentiation and melanisation is still incomplete, and we conclude that expression of Sox10 is rapidly downregulated in differentiating melanocytes in zebrafish.

Studies in mouse have not documented the temporal changes in *Sox10* expression in vivo, but in adult human melanocytes there is evidence that SOX9 expression may partially replace SOX10 and is necessary for maintenance of melanocyte differentiation [30]. Strikingly, studies of cultured differentiating human melanoblasts show that SOX10 expression is lost in differentiating melanocytes, but that SOX9 expression is upregulated [31]. Neither of the zebrafish orthologues, *sox9a* and *sox9b*, have been reported as expressed in melanocytes [32], [33], [34]. To assess directly whether a similar shift from *sox10* to *sox9* expression might occur in zebrafish melanocytes, we used whole-mount in situ hybridisation to assess *sox9a* and *sox9b* expression in zebrafish embryos, but found no evidence for such expression between 24 hpf and 72 hpf (Figure S1; data not shown). We conclude that in zebrafish embryos, *sox10* expression is lost from differentiating melanocytes, but this is not replaced by *sox9* gene expression.

### A simple model for melanocyte development in zebrafish

**Figure 2:**
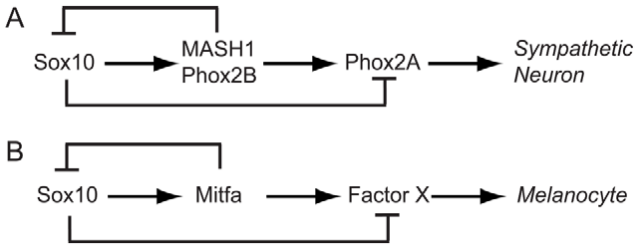
Sox10 function in specification and differentiation of neural crest. A) Sympathetic neuron development, based on [35]. B) Analogous model for melanocyte.

This pattern of *sox10* expression attenuation during neural crest differentiation has also been described for the sympathetic neurons in mouse (Figure 2A; [35]). These authors suggested a model whereby Sox10-mediated activation of MASH-1 and Phox2B drives sympathetic neuron specification, whilst initially feed-forward repression by Sox10 delays sympathetic neuron differentiation; subsequently negative feedback by MASH-1/Phox2B turns off Sox10 and differentiation can now proceed. In melanocyte development, Sox10 drives *mitfa* expression; we have shown in zebrafish that the interaction is direct and identified some of the relevant Sox10-binding sites in the *mitfa* promoter [16]. We asked to what extent the Kim et al. model could be generalised to another neural crest derivative. We proposed an analogous initial model of melanocyte differentiation in which Sox10 drives fate specification by activating *mitfa* expression, but perhaps delayed melanocyte differentiation by a feed-forward repression (Figure 2B). Based on this analogy, we made two predictions. Firstly, melanocyte differentiation genes might be derepressed in *sox10* mutants, just as, in *Sox10* mutant mice, *Phox2A* expression is seen in the absence of MASH-1/Phox2B. Secondly, that *sox10* repression would be directly or indirectly dependent upon *mitfa* expression. Here we explore these predictions experimentally.

### Residual melanin is observed in *sox10* mutant zebrafish embryos

We had previously observed residual melanin in dorsal positions of 3 dpf zebrafish *sox10* mutants, but had not examined this trait in detail [16]. Surprisingly, we had shown genetically that this residual melanin was independent of *mitfa* function; thus, it was consistent with possible derepression of melanocyte differentiation genes in *sox10* mutants. We examined three series of *sox10* mutant embryos, documenting the timing and appearance of these cells (Figure S2). Melanisation in these mutants is substantially delayed compared with wild-type siblings. In contrast to wild-type siblings which showed faint melanin from c. 25 hpf, we were unable to detect melanin before 36 hpf in any of 29 embryos followed (Figure S2C). As in wild-types, numbers of melanised cells increased with developmental age, and tended to form in an anterior-posterior progression (data not shown). Melanised cells were scored for their position with respect to the trunk and tail segments defined by the myotome. The numbers were very variable, with occasional embryos developing melanised cells in up to 21 segments (n = 1), whereas others never showed any (n = 2), and they were usually confined to the trunk and anterior-most tail, and never seen in the posterior-most tail (Figure S2C and data not shown). As noted before, melanin is very faint in these cells, but it undergoes a dynamic change in appearance from initially rather diffuse to later more compacted, forming a tiny but dense spot (Figure S2B). In summary, it seems that melanisation is highly residual and strongly delayed compared with wild-type siblings, consistent with low level derepression of melanogenic genes.

### Melanocyte differentiation gene derepression in *sox10* mutants

**Figure 3:**
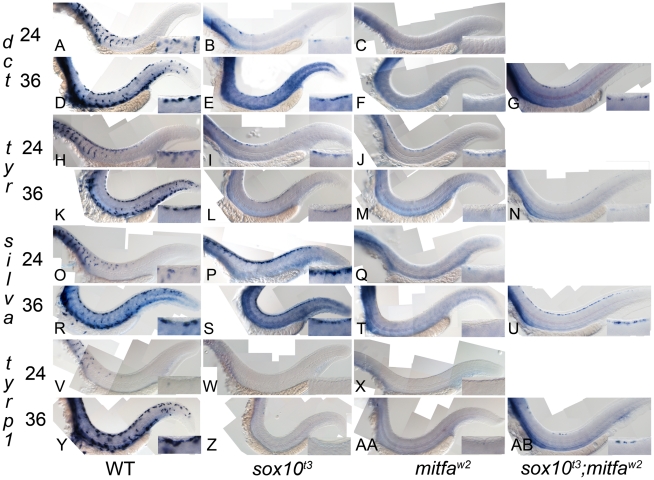
Residual melanocyte marker expression in *sox10*, *mitfa*, and *sox10;mitfa* mutants. A–AB) Expression of *dct*, *tyr*, *silva* and *tyrp1b* in wild-type (WT), *sox10^t3^*, *mitfa^w2^* and *sox10^t3^; mitfa^w2^* mutants is shown at 24 and 36 hpf as indicated. Insets in each panel show enlargement of area of dorsal posterior trunk. Note the pronounced derepression of *silva* and *dct*, mild derepression of *tyr*, and minimal residual expression of *tyrp1*. Note that all in situs were over-developed in order to detect low level expression.

From our model, we predicted that derepression of melanogenic genes would be detected as increased expression in *sox10* mutants compared with *mitfa* mutants, and would be independent of Mitfa function i.e. would persist in *sox10; mitfa* double mutants. We had previously observed residual *dct* expression in dorsally-located cells in *sox10* mutants [36], but had not compared *mitfa* mutants. To assess whether *dct* and other key melanocyte differentiation genes were derepressed in *sox10* mutants compared with *mitfa* mutants, we performed a series of parallel in situ hybridisation studies using four melanogenic genes, *dct*, *tyrp1b*, *tyr* and *silva*, on *sox10* and *mitfa* mutant embryos (Figure 3). Pilot experiments showed that expression in *mitfa* mutants was extremely weak and was undetectable in fish older than 36 hpf, so careful comparisons were made at stages between 24 and 36 hpf (Table 1). In all cases, marker expression in wild-types was very strong, but to test for low level expression in mutants the in situs were stained longer, resulting in higher background than normal. Mutant embryos were developed in parallel with the same probe under identical conditions; expression of all markers in the pigmented retinal epithelium (PRE) was unaffected in each mutant and was used as an internal control for the procedure on each embryo. We saw a consistent pattern for all genes examined, with *sox10* mutants showing slightly more elevated and more consistently-detectable expression (i.e. a higher proportion of embryos showed a signal) and a longer duration (from 24 to 48+ hpf in *sox10* mutants, but from 24 to 30+ hpf in *mitfa* mutants) of detectable expression than *mitfa* mutants (Figure 3 and Table 1). The differential expression of *dct*, *tyr* and *silva* between the two mutants was striking; in contrast effects on *tyrp1b* were subtle, with very little detectable expression being seen (Figure 3), although this residual expression was more consistent and more prolonged (Table 1) in *sox10* mutants.

**Table 1:** Quantitation of numbers of mutant embryos showing residual marker gene expression.

Marker	Genotype	24 hpf	30 hpf	36 hpf	42 hpf	48 hpf	54 hpf	60 hpf
*silva*	*sox10*	**10/10**	**12/12**	**14/14**	**14/14**	**18/20**	12/16	0/11
	*mitfa*	2/6	5/12	0/7	n.d.	n.d.	n.d.	n.d.
*dct*	*sox10*	**14/14**	**12/12**	**14/15**	**8/8**	8/16	1/11	0/12
	*mitfa*	0/6	5/12	0/10	n.d.	n.d.	n.d.	n.d.
*tyrosinase*	*sox10*	**11/11**	**17/17**	**10/11**	15/17	5/13	3/16	0/11
	*mitfa*	**6/6**	**10/10**	0/8	n.d.	n.d.	n.d.	n.d.
*tyrp1b*	*sox10*	**12/12**	**8/8**	7/9	7/7	2/6	0/1	0/8
	*mitfa*	6/9	0/11	0/11	n.d.	n.d.	n.d.	n.d.

Number of homozygous mutant embryos showing detectable expression by in situ hybridisation of named marker gene is given out of total number of mutants examined. Font reflects percentage with residual expression: **90–100%**; 0–89%; n.d., not determined.

As an independent confirmation of these data, we used quantitative real-time PCR on embryos at 30, 36 and 72 hpf (Figure S3). As expected, expression levels of *mitfa*, *dct* and *tyrp1b* are all much reduced in both mutants compared with wild-types. However, consistent with our in situ hybridisation data, at 30 hpf, but not at later stages, the expression levels of *dct*, and to a much lesser extent *tyrp1b*, are significantly higher in *sox10* mutants compared with *mitfa* mutants, confirming the weak and transient derepression of melanogenic genes in the *sox10* mutant embryos.

We had previously shown that residual melanin in *sox10* mutants was not due to low level expression of Mitfa, since *sox10;mitfa* double mutants also showed residual melanisation. To assess whether the low level derepression of melanocyte differentiation genes was also independent of Mitfa, we repeated our in situ hybridisation studies on *sox10;mitfa* double mutants generated by crossing *sox10^+/t3^;mitfa^w2/w2^* parents, so that all embryos were *mitfa* homozygotes, and 25% were double homozygotes. We focused on the 36 hpf stage, when *mitfa* mutants have consistently lost expression, but *sox10* mutants show detectable levels (Table 1); thus, if derepression of melanocyte gene expression was independent of Mitfa, we expected that 25% of embryos would show ‘rescue’ of differentiation gene expression. We found detectable expression of the markers in nearly 25% of embryos (12/60, *dct*; 9/48, *silva*; 9/49, *tyr*; 7/45, *tyrp1b*) from this cross (Figure 3), and interpret these data as showing derepression in most *sox10;mitfa* double mutants. We conclude that melanocyte differentiation gene derepression is independent of *mitfa*.

### Overexpression of Mitfa, but not Sox10, drives precocious expression of melanocyte differentiation genes

**Figure 4:**
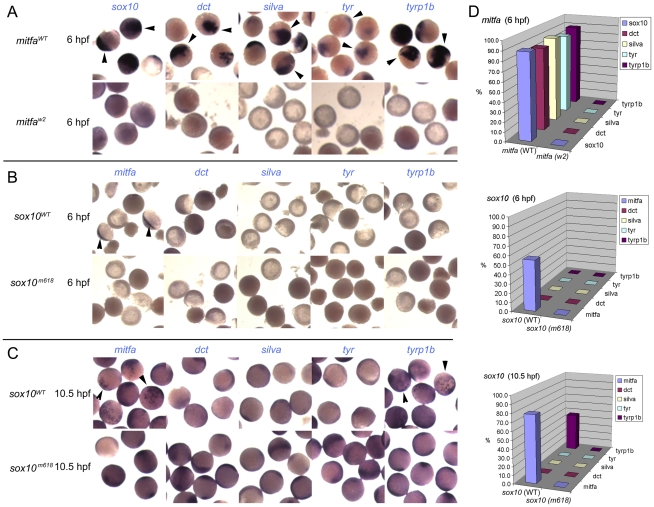
Induction of Mitfa-responsive genes is largely suppressed by Sox10. Representative groups of wild-type embryos are shown after injection of mRNA as indicated to left (*mitfa* (A) and *sox10*, assayed at 6 hpf (B) or 10.5 hpf (C)), raised to stage indicated, then fixed and processed by in situ hybridisation to detect genes named above panels (*purple*). Arrowheads indicate specific signal above background levels. D) Data is quantified as percentage of injected embryos showing expression in graphs at right (n>44 in each case).

To test experimentally the conclusions from this loss of function analysis we performed overexpression experiments in early zebrafish embryos. Embryos were injected at 1-cell stage with 115 pg *sox10* or 35 pg *mitfa* (initial trials showed 115 pg of *mitfa* to induce severe lethality) sense RNA; as controls we used 115 pg of *sox10^m618^* or *mitfa^w2^* RNA respectively which encode the loss of function mutant forms. Injected embryos were examined for induced gene expression by whole-mount in situ hybridisation at an early (6 hpf) or later (10.5 hpf) time-point; note that each of these times is prior to endogenous expression of any of the genes assessed. Mitfa expression might be expected to drive expression of most melanocyte differentiation genes, although data from mouse studies might suggest that Mitf alone may be insufficient for some genes, perhaps especially tyrosinase [24]. In contrast, our Sox10-mediated repression model predicts that Mitfa alone will be sufficient, but that whilst Sox10 alone would drive *mitfa*, Mitfa-dependent expression of other melanocyte differentiation genes (with the likely exception of *tyrp1b*) would be repressed by the presence of Sox10. In all cases, the negative control RNAs induced no gene expression. We observed a clear-cut distinction between the effects of Sox10 and Mitfa overexpression (Figure 4). Overexpression of wild-type *mitfa* mRNA resulted in strong expression of all melanocyte differentiation genes by 6 hpf. In contrast, wild-type *sox10* induced *mitfa*, but no melanocyte differentiation genes, by 6 hpf; by 10.5 hpf, *tyrp1b* was also induced, whereas *dct*, *tyr* and *silva* were not. That this *tyrp1b* expression was Mitfa-dependent was shown by injecting embryos from a cross of homozygous *mitfa^w2^* mutants with *sox10*; whilst *mitfa* transcription was induced by 6 hpf, *tyrp1b* expression was never seen at 10.5 hpf (data not shown). Our results were fully-consistent with our Sox10-mediated repression model with the modification that *tyrp1b* is insensitive to Sox10: Mitfa expression led to melanocyte differentiation gene expression by the early time-point, yet, whilst *sox10* expression induced robust *mitfa* by the early time-point, even at the later one only *tyrp1b* was expressed.

### Co-expression of Sox10 represses Mitfa-dependent expression of melanocyte differentiation genes

**Figure 5:**
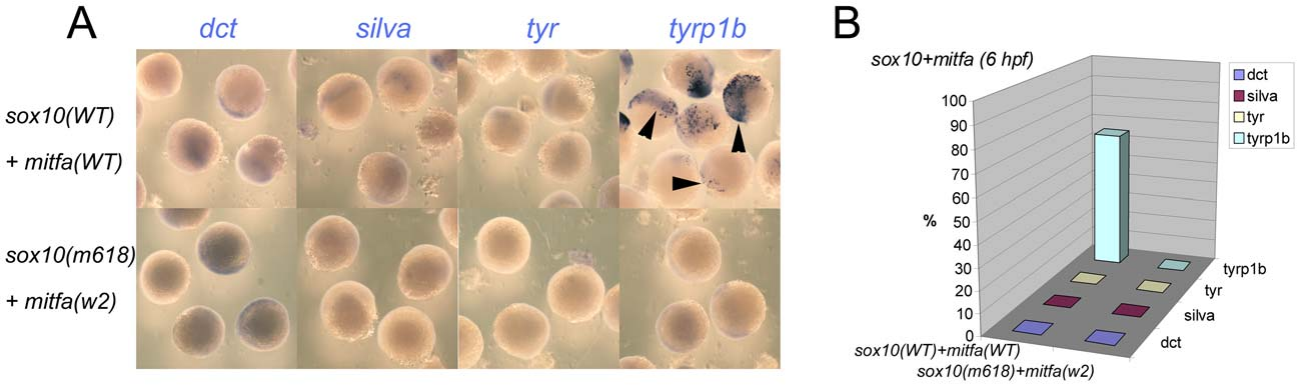
Co-expression of Sox10 and Mitfa represses most Mitfa-dependent expression of melanocyte differentiation genes. A) Representative groups of wild-type embryos injected with *sox10* and *mitfa* mRNA were fixed at 6 hpf and processed for whole-mount in situ hybridisation to detect transcripts of genes indicated. B) Data is quantified as percentage of injected embryos (n>52 in each case).

As a further test of our model, we asked whether co-injection of both *mitfa* and *sox10* RNA would give a Sox10-like pattern of induction, but at the early time-point. Embryos were injected at 1-cell stage with 115 pg *sox10* and 35 pg *mitfa* sense RNA; control embryos were injected with 115 pg of both *sox10^m618^* and *mitfa^w2^* RNA. Again the result was clear-cut; *tyrp1b* expression was readily detected at 6 hpf, whereas *dct*, *silva* and *tyr* were not (Figure 5). We conclude that Sox10 expression can repress the Mitfa-mediated expression of most of the melanocyte differentiation genes tested, but that *tyrp1b* expression is resistant to this effect, and that the timing of *tyrp1b* expression is limited by *mitfa* expression.

### Mitfa regulates *sox10* transcription

**Figure 6:**
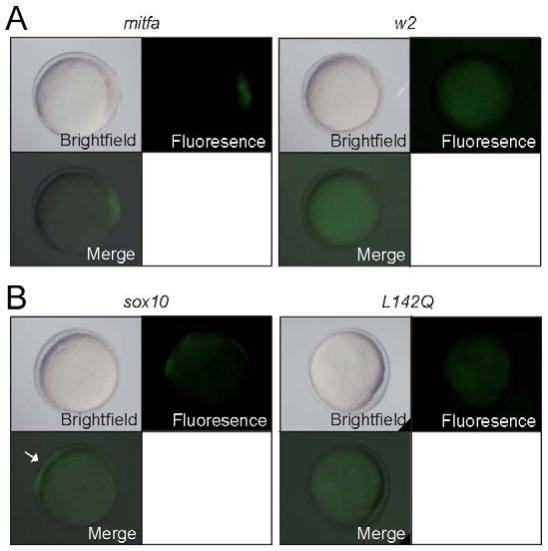
Mitfa-dependent activation of *sox10* expression. Tg(-7.2*sox10:GFP*) embryos were injected with RNA encoding wild-type or *w2* mutant *mitfa* (A) or wild-type or *L142Q* mutant *sox10* (B). Representative embryos are shown at 10.5 hpf, with GFP expression detectable in those injected with wild-type, but not mutant forms. For quantitation, see Table 2.

Our simple melanocyte GRN predicts that loss of Sox10 expression results, directly or indirectly, from expression of Mitfa. There are no published reports of Mitf (positively or negatively) regulating *sox10* expression, but we observed strong transcriptional activation of *sox10* when Mitfa was overexpressed in early zebrafish embryos (Figure 4). This result was surprising since it is in direct contradiction to the predictions of our model, although it does suggest the possibility of Mitfa-mediated *sox10* regulation in vivo. To begin to assess whether this might be direct regulation of the *sox10* promoter by Mitfa, we asked whether GFP was activated in the *Tg(-7.2sox10:GFP)* reporter line, in which a 7.2 kb fragment of the promoter proximal region of sox10 genomic DNA drives GFP expression [37]. In the presence of *mitfa* overexpression, we noted clear GFP expression in transgenic fish at both an early time point (6 hpf), as well as a later (10.5 hpf) one (Figure 6; Table 2), consistent with possible direct regulation.

**Table 2:** Expression of GFP after injection of embryos from *Tg(-7.2sox10:GFP)* outcross.*

*RNA*	6 hpf		10.5 hpf	
*mitfa*	94/257	37%	68/152	45%
*mitfa(w2)*	0/118	0%	0/78	0%
*sox10*	20/319	6%	95/193	49%
*sox10(L142Q)*	0/120	0%	0/80	0%

*NB Only 50% of embryos from this cross would be transgenic, thus maximum percentage GFP^+^ embryos expected is 50%.

In contrast, in the same experiment, very few (6%) embryos injected with *sox10* RNA showed GFP expression at 6 hpf, whereas essentially all transgenic embryos showed GFP by 10.5 hpf, consistent with the idea that Sox10 does not directly regulate this reporter construct, but that Mitfa expression induced by Sox10 can do so. To test this suggestion that *sox10* mRNA only results in expression of the *Tg(-7.2sox10:GFP)* transgene via production of Mitfa, we asked whether expression of the transgene fails in *mitfa* mutant embryos. Thus, we repeated the experiment from Figure 6 in *mitfa* mutant, *Tg(-7.2sox10:GFP)* embryos. As a positive control, we injected *Tg(-7.2sox10:GFP);mitfa^w2/w2^* embryos with *mitfa* RNA; this frequently resulted in GFP expression at both 6 hpf (31/108 (29%) injected embryos) and 10.5 hpf (27/111 (24%)). In contrast, injection of *sox10* mRNA in *mitfa* mutants did not result in GFP expression at either 6 hpf (1/112 (1%)) or 10.5 hpf (0/105 (0%)). Interestingly, these data suggest that, in contrast to *dct* and other differentiation genes, Mitfa-dependent expression of *sox10* is not repressed by the presence of Sox10.

The 7.2 kb of *sox10* regulatory sequences in the *Tg(-7.2sox10:GFP)* transgene contains 6 concensus M boxes, making it plausible that Mitfa binds directly to this promoter. To begin to narrow the region of the *sox10* promoter likely to mediate this response to Mitfa, we repeated these experiments in the *Tg(-4.9sox10:GFP)* line [28] in which the 5′ three M boxes are absent. Interestingly, this transgene shows no response to injected Mitfa at 6 hpf (Figure S4).

**Figure 7:**
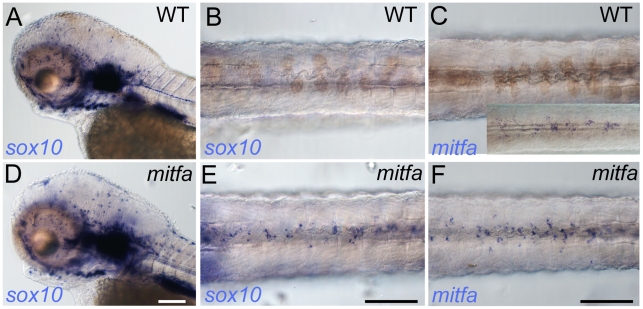
Mitfa-dependent repression of *sox10* expression in neural crest. Whole-mount in situ hybridisation shows prominent expression of *sox10* in peripheral glia and ear in mutants (D) and WT siblings (A); expression in WT melanocytes is undetectable (B), but *mitfa* mutants show prominent expression in many cells in the position of the dorsal stripe (E). (C, F) At this same stage expression of *mitfa* in WT siblings is undetectable under conditions used in this experiment (C), but can be shown by enhancing sensitivity by increasing PTU inhibition of melanisation and extending the signal development time (C, inset). *Mitfa* expression is clearly enhanced in *mitfa* mutants (F). Note that WTs have been treated with 0.00075% PTU to limit melanisation. B,C,E,F) dorsal views of posterior trunk, focused just above spinal cord. Scale bars, 100 µm.

Our data suggest that Mitfa can regulate *sox10* expression, but these experiments, examining the reporter in the context of zebrafish blastomeres, do not necessarily reflect the promoter's response in melanoblasts. To address more directly how *sox10* expression might be regulated by Mitfa in the endogenous situation i.e. in the melanocyte lineage, we examined *sox10* expression in *mitfa* mutants; if Mitfa is necessary for repression of *sox10* we predicted that *mitfa* mutants should show persistent *sox10* expression. We examined *mitfa* mutants at 72 hpf, a stage when wild-type embryos show no detectable *sox10* expression in melanocytes, but show strong expression in the peripheral nervous system and ear (Figure 7A, 7B). In *mitfa* mutants, in addition to the peripheral nervous system expression, we see readily detectable *sox10* expression in the position of the dorsal stripe (Figure 7D, 7E). Furthermore, in *mitfa* mutants we also see a similar pattern of *mitfa* expression in this same region (Figure 7F), strongly suggesting that these cells are neural crest-derived melanocyte precursors that are unable to differentiate due to the lack of functional Mitfa protein. We tentatively conclude that Mitfa can regulate the *sox10* promoter, and that this interaction is likely to have a repressive function in vivo in differentiating melanocytes.

**Figure 8:**
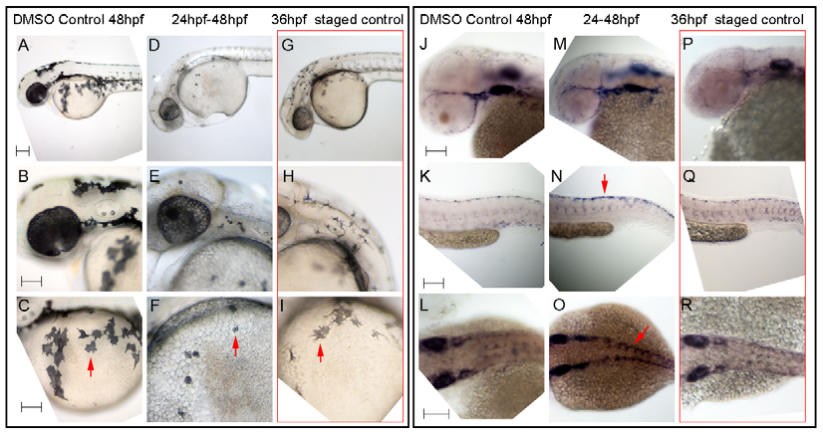
Hdac inhibition with Trichostatin A decreases melanocyte differentiation and prolongs *sox10* expression in neural crest cells. A–I) Live embryos, showing close-ups of head (B,E,H) or yolk sac (C,F,I). (A–C) DMSO control embryo, (D–F) embryos treated with 1 µM Trichostatin A from 24–48 hpf (D–F). Note that whilst all are at 48 hpf nominal age, the Hdac inhibited embryos show morphological retardation, closely resembling 36 hpf untreated fish (G–I). Note that control 36 hpf untreated embryos (G–I) show significantly more melanisation than Hdac inhibitor-treated fish, indicating Hdac inhibition has specific effect on melanisation beyond simply general retardation. J–R) In situ hybridisation with *sox10* probe showing elevated *sox10* expression in premigratory (arrow, N) and migrating neural crest cells of Hdac inhibitor-treated fish (M–O) compared with DMSO controls (J–L). Note that *sox10* expression is elevated even when compared with morphologically-matched 36 hpf embryos (P–R), and is thus not simply an effect of general retardation. Scale bar: 100 µm.

In considering whether any known factors might contribute to this loss of *sox10* expression in melanocytes, we noted the persistence of *sox10* expression described in *colgate/hdac1* mutants [38]. Histone deacetylase1 is a component of multiple complexes that modify chromatin, resulting in selective repression of gene expression. Consistent with the predictions of our model, *hdac1* mutants show both persistent *sox10* expression in neural crest cells and poor melanocyte differentiation, although the connection between these phenotypes was not addressed. To assess whether persistent *sox10* expression in melanocytes was associated with the delay in differentiation, we used chemical inhibition of histone deacetylase function [39] at the time of early melanocyte differentiation, asking whether this resulted in poor melanocyte differentiation and if this correlated with persistence of *sox10* expression. Trichostatin A was applied in each of four time windows: 12–48 hpf, 24–48 hpf, 30–48 hpf and 36–48 hpf. Embryos treated in the 12–48 hpf window showed severe morphological defects, lacking anterior head, but also showed a dramatic reduction in melanocyte pigmentation (data not shown). Those treated from 24–48 hpf again showed severe reductions in melanocyte differentiation (Figure 8D–8F). Although these embryos were of normal morphology, they did appear to show slight retardation, having a morphology similar to approximately 36 hpf embryos. However, comparison of the degree of melanisation of an untreated 36 hpf embryo with the nominally 48 hpf Trichostatin A-treated embryos indicated a clear reduction beyond that expected from delayed development. Later treatment windows showed only weak effects on melanocyte differentiation (data not shown). Using in situ hybridisation we were further able to show that treated embryos showed substantially elevated levels of persistent *sox10* expression in melanocytes (Figure 8N, 8Q). Furthermore, our model requires Hdac-mediated repression of *sox10* expression to be Mitfa-dependent; hence it predicts that Trichostatin A treatment of *mitfa* mutants would not result in further elevation of *sox10* levels above those of untreated *mitfa* mutant controls. An experimental test of this prediction showed that, indeed, *sox10* expression in *mitfa* mutant embryos is not further elevated by Trichostatin A treatment (Figure S5).

Taken together, our data lead us to conclude that repression of *sox10* expression in the melanocyte lineage is both Mitfa-dependent and Hdac-dependent, (most likely mediated by Hdac1 [38]), and that these mechanisms contribute to the differentiation of zebrafish melanocytes in vivo.

### Mathematical modelling and refinement of the melanocyte GRN

Our experimental data was consistent with the major predictions of the simple melanocyte GRN that we had proposed. To assess the GRN more rigorously, and to develop a more quantitative understanding of the model, we turned to mathematical modelling. We constructed a simple dynamical model of the GRN based upon ordinary differential equations, where the transcript concentrations were considered as dynamic variables. Our model aimed to describe the mutual regulation of the genes involved in the GRN by simple activatory and repressive dynamics, and the response of the GRN to external activatory signals, designated Factor A. Studies of both mouse and zebrafish have identified multiple enhancers that drive *sox10* gene expression in neural crest and its derivatives [37], [40], [41], [42], [43]. The factors binding those enhancers are only poorly characterised in both species, but may include Lef/Tcf (downstream of Wnt signalling), Sox9, FoxD3, Pax and AP2. Since this regulation is poorly understood, for the purposes of our modelling we combine these factors into one composite Factor A. It is currently unclear whether in a neural crest cell context these signals are merely transient, or are constantly available. However, given the highly dispersive nature of neural crest cells, we might assume external signals, like Wnt, may be rather transient. Similarly, zebrafish *sox9*, *sox10*, *foxd3*, *pax* and *tfap2* are all downregulated in neural crest cells as they differentiate into melanocytes [34], [44]–[48]; this work). Nevertheless, the data from *mitfa* mutants in Figure 7 indicate that, at least in the vicinity of the dorsal neural tube, one or more components of Factor A remain present at 72 hpf at least. Consequently, for the purposes of our modelling studies, we assumed that Factor A was constant throughout embryonic development.

**Figure 9:**
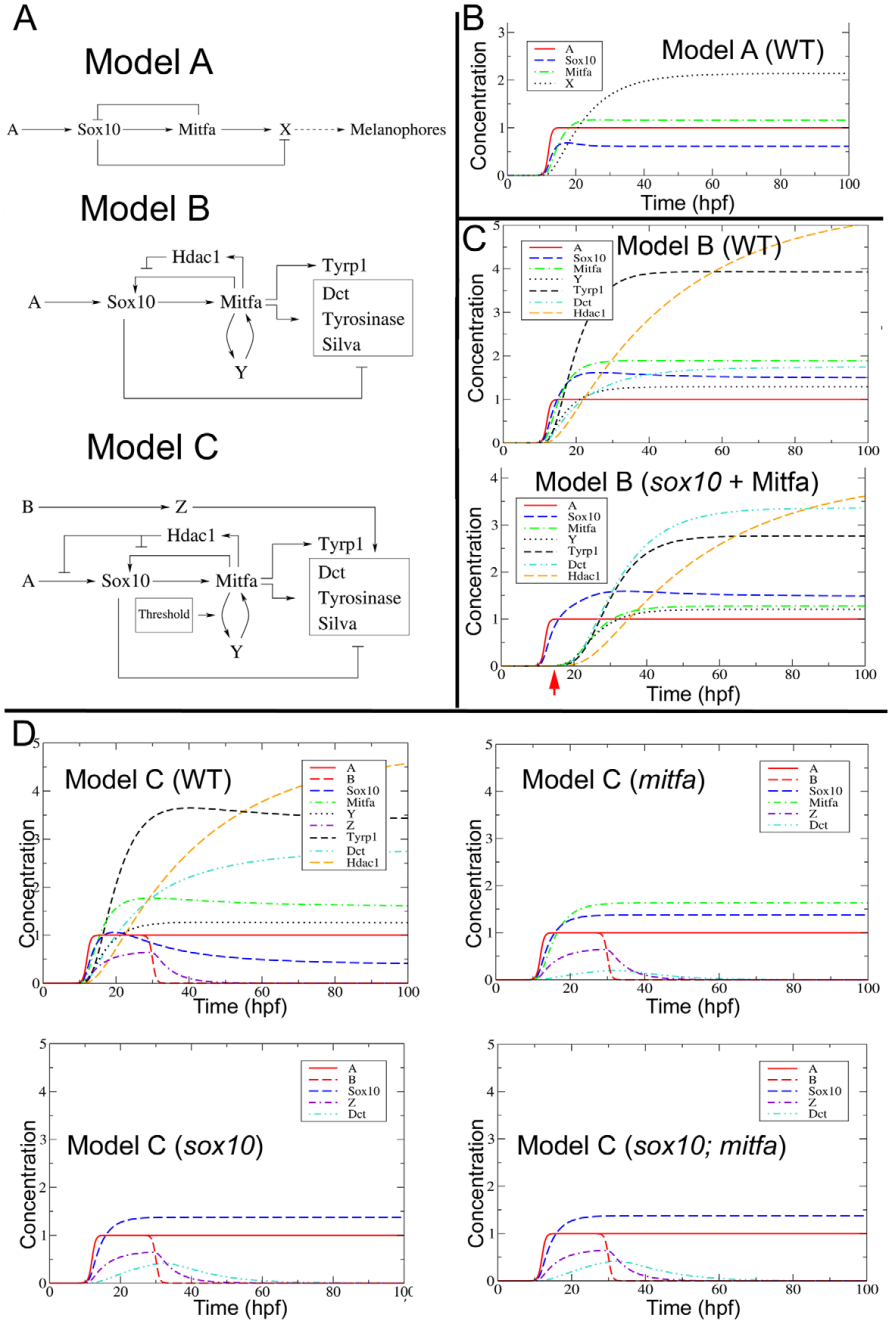
Mathematical modelling and development of the melanocyte GRN. A) Three versions of the melanocyte GRN have been modelled; Models B and C are derived from A, and provide possible solutions to incompatibilities of earlier models with the experimental data. See text for further details. B–D) Simulated output of Model A (B), Model B (C) and Model C (D) in wild-type (WT) embryos or in *mitfa*, *sox10* and *sox10;mitfa* mutants. Lower panel of (C) shows *sox10* mutant in which small amount of Mitfa is provided exogenously (red arrow), mimicking melanocyte rescue experiment (Elworthy et al, 2003 [16]). Graphs plot behaviour of Model for choice of parameters compatible with experimental obervations; shown are changes in gene product concentration (nM) against time (hpf). Parameter values used here are as follows. The initial A and B pulses are characterized by 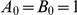
(nM), 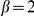
(hrs)^−1^, 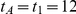 hpf, and 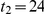 hpf. Furthermore maximal expression levels and degradation rates were fixed respectively at 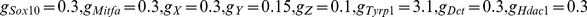 (all values in nM/hrs) and 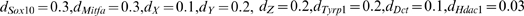 (all values in (hrs)^−1^) in all models. Specific parameters for the different models were the following. Model A: 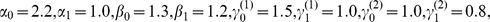 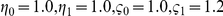. Model B: 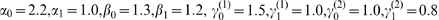 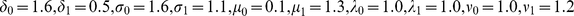, 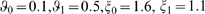. Model C: The same as Model B, with 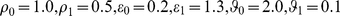. (All values in (nM · hrs)^−1^ for binding constants and in (hrs)^−1^ for unbinding constants respectively.) Other parameters include 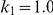
(nM^−1^ hrs^−1^), 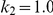
(hrs^−1^), threshold values M* for Mitfa and Y* for Factor Y, M* = Y* = 0.01 (nM).

We explored the rigorous predictions of this initial melanocyte GRN (Model A, Figure 9A) by direct simulation with a widespread exploration of parameter space. Given the lack of quantitative knowledge of most parameters, we restricted ourselves to assessing under which conditions (i.e. parameter value sets) the model predicted i) the long-term maintenance of *mitfa* expression, ii) an initial increase of *sox10*, leading to its maximal expression at intermediate times, and iii) long-term loss (or downregulation, i.e. below a detection threshold) of *sox10* expression, as we have observed in differentiating melanocytes. Direct numerical integration of the ODE system of Model A revealed that the model predicts that both *mitfa* and *sox10* expression are maintained (Figure 9B). However, we found that no parameter settings allowed us to obtain an appreciable difference between *sox10* maximal expression and its steady-state value (see Figure S6), as implied by requirements ii) and iii) above. Maintenance of both *mitfa* and *sox10* arises because the *sox10*-inducing signals (Factor A) are maintained, and these in turn maintain Mitfa expression. Our experimental data above indicates that sox10-inducing signals do seem to persist, at least in the vicinity of the neural tube. However, we note that experimentally, maintenance of Mitfa can be uncoupled from production of Sox10. Our previous study showed that in *sox10* mutant neural crest, transient expression of *mitfa* is sufficient to generate stable (to 5 dpf at least) melanocyte differentiation (Elworthy et al, 2003 [16]). Since this demonstrates that stable melanocyte differentiation can occur in the absence of Sox10 activity if Mitfa is provided even transiently, we rejected Model A as too simplistic. In addition, we noted that it did not incorporate the complexities of Mitfa-mediated regulation of Sox10 as revealed by our experimental studies.

Consequently, we explored the features of a revised model (Model B, Figure 9A) incorporating modifications expected to correct these deficiencies. Firstly, we introduce a Sox10-independent positive feedback loop on Mitfa (Factor Y). Secondly, we add our demonstration that Mitfa-dependent activation of Hdac contributes to the repression of Sox10.

**Figure 10:**
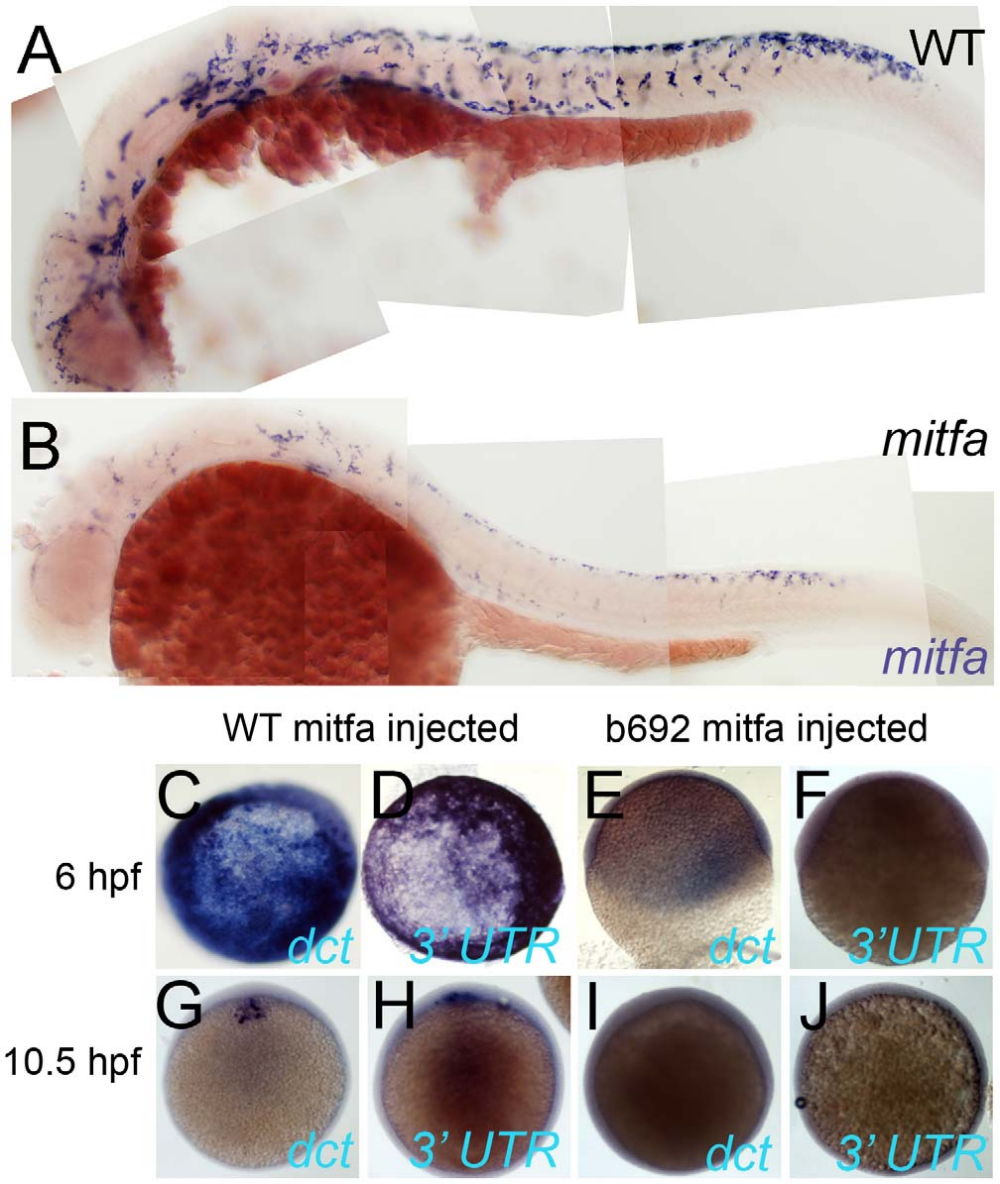
Mitfa-dependent maintenance of *mitfa* expression. A,B) Expression of *Mitfa* is reduced in *mitfa^b692^* mutant. A, B) Embryos from incross of *mitfa* heterozygotes were treated with PTU and processed for in situ hybridization with *mitfa* probes at 30 hpf stage. A majority (53/69; 73%) showed normal strong *mitfa* expression and were presumed wild-type siblings (A, WT), whereas 33/124 (27%) had weakened expression and were presumed *mitfa* mutants (B). C–J) Injection of RNA encoding WT Mitfa drives ectopic expression of *dct* (C,G) and *mitfa* (D,H) at both 6 hpf (C,D) and 10.5 hpf (G,H), whereas RNA encoding the Mitfa(b692) mutant form does not (E,F,I,J). Expression of the endogenous *mitfa* gene is detected using an anti-sense probe corresponding to the 3′ UTR of the gene, a sequence absent from the injected RNA. Scale bar:100 µm.

Model B predicts that in *mitfa* mutant embryos, *mitfa* transcription should be substantially decreased, due to the absence of the positive feedback through Factor Y. In situ hybridisation shows that *mitfa* expression in *mitfa^w2^* mutants is distinctly decreased at 30 and 36 hpf ([13], and data not shown), but given that this mutant results in a premature stop codon, nonsense-mediated decay might also explain the lowered mRNA levels. We thus supplemented these observations with analysis of embryos homozygous for the single amino acid substitution (I121S) allele, *mitfa^b692^* [49]. In these mutants, we again observed an unambiguous substantial reduction in the levels of *mitfa* transcripts in the mutant embryos (Figure 10A, 10B), thus providing support for the biological validity of Factor Y.

Mitfa itself is a clear candidate for Factor Y, and indeed in mouse Mitf functions in conjunction with Lef1 and b-catenin to regulate the *Mitf* promoter [50]. As an initial test whether Mitfa might regulate its own promoter, we asked whether injection of *mitfa* mRNA would induce transcription of the endogenous *mitfa* gene. We used an in situ hybridisation probe for the 3′-UTR of *mitfa*, since the injected mRNA lacks these sequences, as well as examining *dct* induction as a positive control for Mitfa activity. We saw induction of both *dct* and *mitfa* expression upon injection of RNA encoding WT *mitfa* (Figure 10C, 10D, 10G, 10H), whereas neither were seen after injection of RNA encoding either of the Mitfa mutants, Mitfa(b692) or Mitfa(w2) (Figure 10E, 10F; data not shown). We conclude that a Sox10-independent, Mitfa-dependent Factor Y, predicted from mathematical modelling (and perhaps Mitfa itself), is likely to play a major role in maintaining melanocyte differentiation.

Contrary to our intuition, mathematical simulation of Model B showed that this revised model still failed to generate the required downregulation of *sox10* under conditions where *mitfa* was maintained (see Figure S7). Furthermore, it failed to predict two aspects of the phenotype in *sox10* mutant embryos. We found that three further refinements to produce a third model (Model C, Figure 9A) were required for the model to reproduce the experimentally-demonstrated behaviour, as we discuss in the next section.

### Further refinement of the melanocyte GRN is required to explain the wild-type and mutant phenotypes

The first modification required is a change to the way that Hdac1-mediated repression functions on *sox10* expression. In Model B, we postulated that Hdac1 represses Mitfa-dependent *sox10* transcription. However, we found that this was inadequate to allow repression of *sox10* expression in the wild-type (Figure 9C), since constant Factor A persists (Figure S7). In this context, the identification of Hdac as a repressive factor becomes rather striking, since the effects of deacetylation might be expected to affect multiple enhancer elements. As we have noted experimentally, *sox10* expression is repressed in differentiating melanocytes, so in Model C we show Hdac as repressing Factor A-dependent *sox10* expression, as well as Mitfa-dependent activation of sox10 transcription (Figure 9A). This model now reproduces the wild-type observations (Figure 9D and Figure S8).

Secondly, we found that it is crucial to incorporate a threshold response within the Factor Y-mediated feedback in Model B. In the absence of such a threshold, the positive feedback of Factor Y ensures that in *sox10* mutants the absence of melanocyte differentiation is only an *unstable* state associated with [*mitfa*] = 0, since even the lowest level expression of *mitfa* would be expected to trigger positive feedback leading to high level *mitfa* expression and subsequent melanocyte differentiation (Figure 9C, *sox10*+Mitfa). The biological observations are unambiguous – even vaguely normal looking melanocytes are exceptionally rare in *sox10* mutants (RNK, pers. obs.) – suggesting that the positive feedback loop with Factor Y must exhibit threshold behaviour, so that the [mitfa] = 0 state is stabilised at low levels of Mitfa or of Y. In both *sox10* and *mitfa* mutants expressing Mitfa under the *sox10* promoter, melanocyte rescue is relatively unlikely (70% of embryos show no melanocytes, and most embryos showing rescue show <10 melanocytes per embryo [16]), but when it does occur melanocyte morphology and differentiation appear normal, consistent with the GRN being bistable. To account for this behaviour, we have incorporated a threshold response to the Factor Y feedback loop.

Thirdly, Model B failed to predict the low level derepression of melanocyte differentiation genes in *sox10* or *sox10;mitfa* double mutants (data not shown). One solution to this problem, a Sox10-independent Factor Z driving (low level) expression of melanocyte differentiation genes, is incorporated into Model C (Figure 9A). Our efforts to model Factor Z initially assumed that it was driven by Factor A, and thus remained constant. However, under these assumptions, we were unable to reproduce the very weak and transient expression of differentiation genes observed experimentally. Instead, we made the assumption that Factor Z is activated by an unknown Factor B, and Factor B is only transiently expressed in the melanocyte lineage. Mathematical exploration of this model shows that, whilst the non-zero wild-type steady state seen before in Model B is conserved, Model C also reproduces the gene expression patterns seen in *sox10*, *mitfa* and *sox10;mitfa* mutants (Figure 9D). In particular, expression of *dct* (representing the melanocyte differentiation genes repressed by Sox10) is seen at low levels in *mitfa*, *sox10* and *sox10;mitfa* mutants, but this is weakest and most transient in *mitfa* mutants.

### Sox9b has the properties of Factor Z

This modelling is only useful in so far as it allows us to correctly predict novel features of the biology. We chose to explore candidates for Factor Z. Such genes would have no prominent role in wild-type melanocytes (i.e. loss of gene function would not have a melanisation defect), but they would need to be expressed in neural crest cells and to drive low level melanisation in *sox10* mutants; in addition they would be only transiently expressed in melanocyte progenitors.

**Figure 11:**
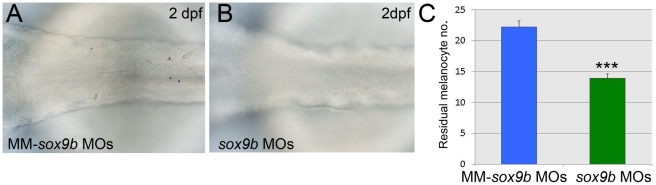
Sox9b is a component of the melanocyte GRN and shows properties consistent with Factor Z. Expression of residual melanin is compared in *sox10* mutant embryos treated with *sox9b* morpholinos (B, *sox9b*MOs) or with control 5 bp mismatch morpholinos (A, MM-*sox9b* MOs). C) Quantitation confirms that weak residual melanin is significantly reduced by Sox9b knockdown compared to treatment with mismatch morpholinos. Graph shows mean±s.e.m., n = 154 (MM-*sox9b* MOs), 159 (*sox9b*MOs). ***, p<0.0001.

In adult human melanocytes SOX9 is likely to regulate DCT [30]. There are two zebrafish orthologues of SOX9, but neither *sox9a* nor *sox9b* nor *sox9a;sox9b* mutants show a loss of melanisation [34]. Unlike *sox9a*, *sox9b* is expressed in early neural crest cells, but then is downregulated ahead of *sox10* in progenitors for all except craniofacial cartilage (data not shown; [34]). We used previously published *sox9b* morpholinos [51] to address whether morpholino-mediated knockdown of Sox9b would result in loss of residual melanin in *sox10* mutants (Figure 11). The numbers of residual melanised cells in *sox10* mutants at 2 days post fertilisation (dpf) was significantly reduced in embryos injected with 0.5 ng of each *sox9b* morpholino compared with embryos injected with sox9b mismatch morpholinos (Figure 11A–11C). We deduce that Sox9b can drive Sox10 and Mitfa-independent melanisation displayed by *sox10* mutants.

We conclude that Sox9b shows the characteristics predicted for Factor Z and that it at least contributes to this role in zebrafish. Furthermore, our data provides biological validation of Factor Z, a second feature of the melanocyte GRN predicted as a result of the mathematical modelling. We also note the transient expression of sox9b in NCCs, broadly consistent with our deductions from the modelling above.

## Discussion

**Figure 12:**
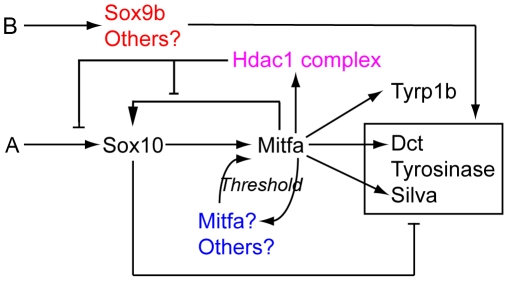
Revised melanocyte GRN derived from this study. Components of Factor Y (blue) and Z (red) are indicated. We propose that one or more components of Hdac1-containing repression complex is regulated by Mitfa, and mediates Mitfa-dependent repression of *sox10* expression.

In this study we have used a combination of genetic experimentation and mathematical modelling to build upon our initial description of melanocyte specification under the control of Sox10 [16]. We have considerably expanded and refined the GRN associated with melanocyte specification and differentiation in embryonic zebrafish (Figure 12). We have shown multiple new features, including 1) Sox10-mediated repression of many Mitfa target genes; 2) the transient nature of Sox10 expression in differentiating melanocytes, resulting from 3) Mitfa-dependent repression of Sox10, likely via 4) a mechanism involving Hdac1 complex; and 5) Sox10-independent weak activation of melanogenesis genes.

An early comparison of the core GRN of melanocytes in mouse and zebrafish had concluded that they were evolutionarily divergent [24]. That comparison focused on a basic description of the role of Sox10 in melanocyte differentiation, noting that in zebrafish there was no requirement beyond melanocyte specification (i.e. activation of *mitfa*), whereas it was required positively both for melanocyte specification (*Mitf* expression) and differentiation (*Tyr* expression) in mouse. The more extensive examination of the zebrafish GRN presented here both supports the suggestion of some evolutionary divergence in the role of Sox10, but also identifies a series of new features that will need to be examined in the mouse system.

The data in the Hou et al study show that Mitf is not sufficient to rescue melanisation in *Sox10* mutant neural crest cells, at least in primary cultures of neural crest cells, since Sox10 function is also required to drive *Tyr* expression [24]. Our data validate our previous conclusion that *ongoing* Sox10 function is not necessary for melanocyte differentiation in zebrafish *in vivo*, since *mitfa* expression in early neural crest cells was sufficient to fully rescue melanocyte differentiation, even up to 5 dpf [16]. However, we now show that Sox10 *does* have a role beyond melanocyte specification (i.e. transcriptional activation of *mitfa*), although it appears to be purely repressive. Certainly, the effects of Sox10 on *Tyr* expression in mouse (synergistic activation with Mitf) and zebrafish (antagonistic repression) are in stark contrast. These data now make untenable the conclusion reached by Hou et al that the differences in the role of Sox10 might explain the differences in timing of melanisation in mammals (late) and fish (early) [24]. Further work to define in much greater detail the melanocyte GRN in each species will allow identification of the key differences between them actually controlling the distinctive timing of melanisation.

Our observations in zebrafish beg the question of whether there is Sox10-dependent repression of melanocyte genes *in vivo* in mouse. Such studies are hindered by issues of sensitivity of whole mount in situ hybridization and the difficulties of directly comparing gene expression levels in melanocytes of wild-type and mutant strains, but one recent paper attempts to standardise the analysis of gene expression for multiple melanocyte markers in E11.5 mouse embryos. Using their semi-quantitative scoring system, *Gpnmb* (but not *Dct, Si, or Tyr*) expression is detectable in *Sox10^LacZ/LacZ^* mutants but not in *Mitf^Mi/Mi^* mutants [52], providing a hint that Sox10-dependent repression of melanocyte differentiation genes may occur in mouse.

It certainly seems surprising that two homologous cell-types, with striking conserved phenotypic characteristics, might show such a substantial change in their GRN. Comparison of GRNs in an evolutionary context is still in its infancy, but already examples of substantial differences between the circuitry of homologous cell-types are known. For example, in echinoderm development, conserved gene expression in homologous domains of sea urchins and sea stars often results from divergent regulatory inputs i.e. the output is conserved, but the regulatory mechanism has diverged [53]. Conceptually, it is trivial to imagine how mutations in regions near the binding site of an activatory transcription factor might allow binding of a co-repressor at that promoter. It will be exciting to identify the molecular basis for the change in Sox10 function.

But what might be the biological function of the Feed-Forward Repression by Sox10? In the mouse sympathetic neuron, Kim et al suggest that this circuitry delays differentiation and maintains multipotency [35]. Delay of melanocyte differentiation and maintenance of progenitor multipotency is an attractive hypothesis in the zebrafish too. Recent study of an *mitfa:GFP* transgenic line indicates that not all neural crest cells that turn on *mitfa* will become melanocytes, since some will form iridophores instead (Curran et al., 2010). Thus, in zebrafish expression of *mitfa* does not represent commitment to the melanocyte lineage; the Feed-Forward Repression loop we have defined might contribute to that maintenance of multipotency in the early melanocyte precursor. Loss of Sox10 expression would then be necessary for commitment to a differentiated state. In this context, it is intriguing that mouse melanocytes, which retain *Sox10* expression, appear to have also retained multipotency, which can be exhibited when isolated and cultured [54].

We have proposed that Sox10 functions to delay melanocyte differentiation in embryonic zebrafish. Likewise, a similar conclusion was reached for the role of Pax3 in adult mouse melanocyte stem cell differentiation. Thus Lang and colleagues demonstrated that Pax3 acted with Sox10 to drive transcription of *Mitf*, whilst feed-forward repression by Pax3 delayed expression of *dct* [55]. Pax3 morphants are not described as having a dramatic melanocyte differentiation phenotype, but the detailed timing of melanocyte differentiation was not examined [44]. Our initial investigations using Pax3 morpholinos (MN and RNK, data not shown) have failed to detect an effect on either wild-type or *sox10* mutant melanogenesis, so it remains unclear whether the role for Pax3 is conserved in fish.

One key feature of the zebrafish melanocyte GRN that we have uncovered is the rapid down-regulation of *sox10* during early differentiation. A major task will be to elucidate the molecular basis for this. Our study only begins to address this issue, indicating that *sox10* repression in melanocytes is Mitfa-dependent, but leaves open whether *sox10* is a direct target of Mitfa. Development of further tools for the zebrafish, especially good antibodies for Mitfa to allow ChIP-chip or ChIP-seq studies, will allow this important question to be addressed definitively. Our initial data provide a strong hint that the effect of Mitf, whether direct or indirect, on *sox10* is highly context dependent; Mitfa activates the *sox10* promoter in the context of embryonic blastomeres, whereas it represses the same promoter in the context of melanoblasts. We note that the 7.2 kb genomic DNA fragment in the *Tg(-7.2sox10:GFP)* reporter that responds to Mitfa contains 6 consensus M boxes, whereas 3 of these are missing in the *Tg(-4.9sox10:GFP)* that does not [56]. Testing whether Mitfa directly regulates *sox10* in vivo via one or more of the 5′ M boxes is a priority for future work.

We hypothesize that the presence of a repressive cofactor in melanoblasts alters the effect of Mitfa on the *sox10* promoter. Little is known of repressive cofactors in zebrafish melanocyte development. Zebrafish *histone deacetylase1/colgate (hdac1/col)* mutants showed delayed melanocyte differentiation; whilst *sox10* expression in early neural crest was indistinguishable from wild-type, *sox10* expression was prolonged to at least 52 hpf, although it was unclear if these phenotypes were causally linked [38]. We have shown here that chemical inhibition of Hdac function during the phase of early melanocyte differentiation results in prolonged *sox10* expression in differentiating neural crest cells, and in impaired melanogenesis. This is strikingly consistent with the core GRN we have identified here, and supports the hypothesis that Mitfa-dependent repression of *sox10* requires Hdac1. However *hdac1* expression is both maternal and zygotic [57], so transcriptional regulation of *hdac1* itself by Mitfa is unlikely to explain the repression of *sox10* in differentiating melanocytes. We speculate that Mitfa may regulate recruitment to the *sox10* promoter of an Hdac1 complex [58], resulting in deacetylation of this chromatin and repression of *sox10* transcription.

The identification of Mitfa-dependent activation of the Hdac complex proved crucial to explain the repression of *sox10* transcription. In our modelling we initially assumed that Mitfa-dependent repression affected only the regulation by Mitfa itself, switching it from an activator to a repressor. However, modelling the GRN in this way proved ineffective, because it failed to shut-down *sox10* transcription, apparently due to the fact that whilst the Mitfa influence was repressed, input from Factor A persisted, and hence Factor A-dependent expression became dominant. The realization that Hdac complex mediated the Mitfa-dependent repression immediately provided a resolution to this problem, since deacetylation would be expected to repress activity of many/all enhancers of the *sox10* gene, making it likely that Factor A-dependent *sox10* expression, as well as Mitfa-dependent expression, would be inactivated in the wild-type situation. In contrast, in the *mitfa* mutant situation, Factor A remains, so that we see persistent *sox10* and *mitfa* expression, just as observed in vivo. Satisfyingly, this was exactly the behaviour we saw when we modeled the GRN in the light of this insight. Hence, whilst the presence of Factor A seems to persist, as revealed by the *mitfa* mutant phenotype, our intuition that the *influence* of Factor A would be transient in the wild-type situation appears to be well-founded, resulting from the global shut-down of *sox10* transcription mediated by Hdac complex.

We have demonstrated for the first time that in the presence of Sox10, many Mitfa-mediated transcriptional responses are repressed. At first glance, it is surprising therefore that when we over-express Mitfa in zebrafish blastomeres, melanocyte differentiation genes are expressed robustly, despite the observation that *sox10* is also expressed. We propose that the explanation lies in the timing of expression of Sox10 *protein*. When *sox10* mRNA is injected alone, Sox10 protein forms before *mitfa* can be transcribed. Thus, Mitfa protein is functioning in the context of substantial amounts of Sox10; in contrast, when *mitfa* is expressed alone, Mitfa protein is functioning before *sox10* transcription and hence is working in the absence of Sox10 protein. The test of this is the coinjection of both *sox10* and *mitfa* mRNAs; in this context both Sox10 and Mitfa proteins would be formed together and hence again Mitfa would be functioning in the context of Sox10 protein. The prediction is that melanocyte differentiation genes would be repressed; this prediction is directly borne out by our experimental test (Figure 5). We conclude that our data is, in fact, consistent in suggesting that Sox10 represses Mitfa-mediated melanocyte differentiation.

Nonetheless melanocyte differentiation in vivo occurs whilst *sox10* transcripts remain detectable (Figure 1). We propose that, in part, the explanation lies in Sox10-mediated repression depending more on the *ratio* of Sox10:Mitfa proteins: our preliminary data exploring the effects of changed ratios of *sox10:mitfa* supports this [56]. In mouse sympathetic neuron differentiation, *Sox10* heterozygotes show derepression of Phox2A, but normal expression of MASH1 and Phox2B, indicating that here higher levels of Sox10 are required for repression of differentiation than for specification [35]. In addition, the explanation likely lies in the complex integration of multiple factors as inputs on melanocyte differentiation gene expression. Thus, here we have identified Sox9b as an unexpected factor driving melanocyte differentiation. Given that, as we show here, *sox9b* expression is not detectable in differentiating melanocytes, this role must be transient, and restricted to the early phase of melanocyte development. Whilst melanisation is consistently repressed in *sox10* mutants injected with *sox9b* morpholinos, effects on residual *dct* expression were more variable; whereas *sox10* mutant embryos injected with the mismatch morpholino showed low level *dct* expression, this expression was sometimes reduced in *sox9b* morphant;*sox10* mutant embryos, although not statistically significant overall (MN and RNK, data not shown). We suggest that at these early stages of melanocyte differentiation, *dct* expression reflects the integration of multiple activatory (Mitfa, Sox9b, others?) and inhibitory (Sox10, others?) inputs. Our mathematical modelling here (Figure 9D) shows that this scenario can generate a convincing reproduction of our semi-quantitative in situ observations. The challenge for the future will be in vivo quantitation of the various key parameters of the model in order to examine how precisely the model and the in vivo situation match each other.

Our mathematical modelling approach, used iteratively with experimental data, has made specific predictions about the properties of currently unidentified factors in melanocyte differentiation. Importantly, we illustrate the power of our systems biology approach by experimentally identifying Sox9b as a factor fulfilling the properties of Factor Z. Our data here on melanocytes extends the evidence for partial redundancy of Sox10 and Sox9b in neural crest development initially shown for sensory neurons [28]. Indeed, we noticed that *sox9b* morphants also show significantly reduced numbers of ‘escaper’ iridophores too (MN and RNK, data not shown), suggesting this partial redundancy between these closely-related transcription factors may be a general feature.

Our modelling also implied the activity of a Sox10-independent, Mitf-dependent transcriptional activator of Mitfa, Factor Y, providing a positive feedback loop to allow stable melanocyte differentiation. We demonstrate that in *mitfa* mutant zebrafish embryos, *mitfa* expression is reduced compared with wild-type siblings consistent with our suggestion of a role for Mitfa in maintaining *mitfa* expression. Consistent with this, we also show that overexpression of Mitfa results in rapid, precocious expression of the endogenous *mitfa* gene. Likewise, while Mitf expression in mouse E11.5 embryos is prominent throughout the body, in *Mitf^Mi/Mi^* mutants it is weakened and only detectable in the tail, the developmentally youngest region [52]. These data strongly support the suggestion from our modelling that maintenance of *mitfa* expression is (directly or indirectly) dependent upon Mitfa function, and that this feedback is conserved in mouse melanocytes too.

Apart from Sox10, several other transcription factors have been shown to regulate *Mitf* [59]. One candidate for Factor Y is CREB, acting downstream of elevated cAMP induced by Melanocyte Stimulating Hormone (MSH)/Melanocortin Receptor 1 (Mc1R) signalling [60]. MSH has a clear role in background adaptation, and Mc1R expression is maintained throughout embryonic development [61], [62]. However, current evidence for the role of Mc1R in melanisation in zebrafish based on morpholino knockdown is conflicting [63], [64]. In our attempts to reproduce these morpholino studies we saw a transient decrease in melanisation, consistent with [63], but this seemed to be in large part due to embryonic retardation, indicating that, in agreement with [64], Mc1R signalling in zebrafish is unlikely to play a major role in melanocyte melanisation (LV and RNK, data not shown). We conclude that Mc1R signalling is not likely to contribute to Factor Y, at least in the embryonic melanocytes.

Understanding the mechanisms stabilizing the differentiated melanocyte fate is likely to have particular relevance for our understanding of melanoma. Levels of the steady state activity of Mitf appear to be crucial to the melanoma phenotype, with high Mitf activity associated with differentiation and lowered levels with proliferation and melanoma [65]. Several factors identified as regulating *Mitf* in development, also play major roles in melanoma; for example, WNT/b-catenin dependent regulation of MITF transcription has been demonstrated by chromatin immunoprecipitation and plays a major role in the transformed phenotype by promoting both proliferation and survival of melanoma cells [66]. A mouse melanoma model generated by combining melanocyte-specific expression of both constitutively active β-catenin and activated N-ras generates frequent melanomas [67]. In the context of our work, it is interesting that melanocytes from this strain frequently become immortalised, and do not fully pigment [67].

In conclusion, our systems biology approach has identified several new and unexpected features to the core GRN underlying melanocyte specification and differentiation *in vivo*. We have demonstrated a role for Sox10 in antagonising Mitfa-dependent differentiation; have firstly predicted, then identified Sox9b as part of, a factor with a transient role in Mitfa-independent melanisation observed in *sox10* and *sox10;mitfa* mutants; have predicted and then shown that *mitfa* expression is, directly or indirectly, Mitfa-dependent; and have provided the first indication that Mitfa might negatively regulate *sox10* expression in differentiating melanocytes. Both the latter mechanisms are likely to be major factors stabilising differentiation of melanocytes in zebrafish. The stage is now set for a comprehensive analysis of the zebrafish melanocyte GRN, by incorporation into the model of other known and unknown regulatory functions combined with a network analysis of the motifs identified therein, in order to truly understand the basis for stable differentiation of this medically-important cell-type. We suggest that application of our approach to other medically-important cell-types is likely to be valuable.

## Materials and Methods

### Ethics statement

This study was performed with the approval of the University of Bath ethics committee and in full accordance with the Animals (Scientific Procedures) Act 1986.

### Fish husbandry

Embryos were obtained from natural crosses and staged according to Kimmel et al. [68]. We used the *sox10^t3^* allele [29], the *mitfa^w2^* [13] allele except where stated otherwise, when we used *mitfa^b692^* [49], and the *Tg(-4725sox10:GFP)^ba3^* and *Tg(-4.9sox10:EGFP)^ba2^* lines [28], [37].

### In situ hybridisation and antibody staining

RNA in situ hybridization was performed according to Thisse et al. [69], except probes were not hydrolysed and embryos were incubated at 68°C in hybridization steps. Probes used were *sox10* [15], *dct* [36], *mitfa* [13], *silva* (ZIRC cb397; [70], *tyrosinase* [71], *tyrp1b* (clone number 6894514 from Geneservice, GenBank reference CB353867, subcloned as an EcoRI/XhoI fragment into Bluescript), *gch* [72], *xdh* [72] and *paics* (Plasmid and probe generated by T. Chipperfield and C. Nelson).

Antibody staining with anti-Sox10 (1∶10000, [73]) and Alexa Fluor 488 (1∶2000, Invitrogen, A21206) was performed largely as Ungos et al. [74].

Embryos were viewed using an Eclipse E800 (Nikon) using either DIC or fluorescence microscopy as appropriate. Embryos were scored for Sox10 and *sox10* expression by scoring 20 pigmented melanocytes in each of 5 embryos at each time point.

### RNA injection

One cell stage embryos were injected with RNA using standard methods as in Dutton et al. [15]. RNA was produced and recovered using the mMESSAGE mMACHINE and MEGAclear kits (Ambion) from hs>*sox10* and hs>*sox10^m618^* templates linearized with Asp718 [15] or CS2+*mitfa^WT^* and CS2+*mitfa^w2^* linearised with Not1 [13]. *sox10*, *sox10^m618^* and *mitfa^w2^* RNA were diluted to a concentration of 25 ng/µl, *mitfa* RNA was diluted to 6.25 ng/µl including 0.0005% Phenol Red. Embryos were injected with 4.6 nl RNA and grown for 6 or 10.5 hours at 28.5°C. Embryos were then processed for in situ hybridisation or scored for GFP fluorescence using an MZ12 dissecting microscope (Leica).

### Promoter analysis

DNA sequence was submitted to TRANSFAC public version 6.0 using the Pattern Search for Transcription Factor Binding Sites (PATCH 1.0) interface. Parameters were set to look for vertebrate transcription factor binding sites of 6 bp or more with the maximum number of mismatches being set at zero [75].

### Chemical inhibition

Trichostatin A (TSA, [R-(E,E)]-7-[4-(Dimethylamino)phenyl]-N-hydroxy-4,6-dimethyl-7-oxo-2,4-heptadienamide)(Sigma-Aldrich) was kept as a 5 mM in DMSO stock solution (0.2 µm-filtered) at −20C. Batches of embryos were treated with 1 µM Trichostatin A in Petri dishes, during each of four time windows (from 12 hpf to 48 hpf, from 24 hpf to 48 hpf, from 30 hpf to 48 hpf and from 36 hpf to 48 hpf) at 28.5°C. Control embryos received equivalent doses of DMSO alone. Melanocyte phenotypes of live embryos were documented at 48 hpf under a Nikon E800 microscope; embryos were anesthetized with Tricaine (Sigma-Aldrich) and mounted on slides under coverslips in 30% methylcellulose.

### Quantitative real-time PCR

RNA was extracted from samples of 40 embryos of each genotype (decapitated after anaesthesis with Tricaine) using TRIREAGENT (Sigma-Aldrich, T9424). First strand cDNA was synthesized using the Invitrogen First strand cDNA synthesis kit with Superscript III and random hexamers. Real time quantitative PCR was performed in duplicate using SYBR Green I PCR Master Mix (Roche) and a Lightcycler II machine according to the manufacturer's instructions. Primers were designed spanning an intron using Primer3 Plus software (http://www.bioinformatics.nl/cgi-bin/primer3plus/primer3plus.cgi.). The following primers were used: *gapdh*: forward ACCAACTGCCTGGCTCCT, reverse TACTTTGCCTACAGCCTTGG; *mitfa*: forward CTGGACCATGTGGCAAGTTT, reverse GAGGTTGTGGTTGTCCTTCT; *dct*: forward TCTTCCCACCTGTGACCAAT, reverse CTGATGTGTCCAGCTCTCCA; *trp1b*: forward CGACAACCTGGGATACACCT, reverse AACCAGCACCACTGCAACTA. Gene expression was normalized against zebrafish *gapdh* expression in wild-type embryos. Quantitative RT-PCR data were analysed using the (ΔΔCt) method [76]. Student's t-test with Bonferroni correction for multiple comparison were performed using GraphPadPrism 5.0 to test the null hypothesis that there was no significant difference in gene expression levels between *mitfa* and *sox10* mutants. In all tests, difference was considered significant if p<0.017.

### Mathematical modelling

We constructed a mathematical model for gene regulation as a one stage process: binding and unbinding of transcription factors (TFs) to DNA was assumed to regulate protein production in a single step of synthesis, without explicit modelling of intermediate mRNA levels. The model was expressed in terms of a system of ordinary differential equations (ODEs). Binding and unbinding of TFs were described as faster processes than protein synthesis and degradation. This allowed us to solve the transcript dynamics in conditions of quasi-equilibrium for the TFs. The result was a description of both activatory and repressive regulation in terms of Hill-like functions. By using appropriate combinations of Hill functions, Models A, B and C (Figure 9A) were then described mathematically. The derivation is presented in the accompanying Text S1.

Models were investigated by direct numerical integration and, in the case of Model C for the *sox10* mutant, by steady-state analysis. The steady-state analysis was obtained by setting time derivatives to zero, and by solving analytically the corresponding set of algebraic equations. This gave information about the long time behaviour of this GRN, and allowed us to draw conclusions independent of the particular set of chosen parameters. The time-dependent solution was computed numerically by using a standard finite differences algorithm (Euler). Parameter values were chosen so as to reproduce the sought behavior, constrained by available experimental evidence whenever possible. For instance, knowledge about typical time scales of the relevant concentrations fixed gene expression and decay rates.

Furthermore, the robustness of our conclusions with respect to the chosen parameter values was assessed by plotting the steady state value of Mitfa, and the steady state and the maximal values of Sox10, as functions of the different activatory and repressive regulations between mitfa and sox10 in all studied models (see Figures S6, S7, S8). Here our aim was not to identify a unique parameter set that reproduced the experimental data, but rather to assess to what extent our conclusions might be broadly independent of the specifically chosen parameters. In this sense our results should be taken as qualitative, given the lack of knowledge of most parameter values, but still representative of typical dynamical behavior.

## Supporting Information

Figure S1 Neither *sox9a* nor *sox9b* are expressed in differentiated melanocytes. Lateral views of whole embryos (left) and dorsal views of dorsal stripe region (insets right, location indicated by lettered bars) show 60 hpf (A,C) and 72 hpf (B, D) embryos. In C, inset b shows a deeper focal plane than that in inset a. Embryos were treated with PTU to allow detection of even very weak signals. Scale bar 100 µm.
(TIF)

Figure S2 Residual melanised cells in *sox10* mutants appear late and then increase with time. A) Photographs of dorsal trunk of a single embryo showing dynamic changes in residual melanised cells. Note how initially many cells show diffuse melanin (arrows) and how new melanised cells appear with time (arrowheads). B) Photographs of single melanised cell at consecutive time-points, showing change from diffuse melanin (41 hpf) to tiny, dense spot (43 hpf). C) Graphical plot of mean±s.e. number of segments containing residual melanised cells from a typical series of embryos (n = 19).
(TIF)

Figure S3 Quantitative RT-PCR of *mitfa*, *dct* and *trp1b* expression in wild-type (WT), *mitfa^w2^* mutants and *sox10^t3^* mutants. Values shown are mean±s.d. at 30 hpf, 36 hpf and 72 hpf. Expression levels in WT controls were normalised to GAPDH for each sample, and expression is shown as percentage of WT transcript expression levels normalised to GAPDH. Expression levels that were statistically significantly elevated in *sox10* mutants compared with *mitfa* mutants are indicated (1-tailed t-test with Bonferroni correction for multiple comparisons, **).
(TIF)

Figure S4 Mitfa-dependent regulation of *sox10* transgenes narrows candidate regulatory elements. RNA encoding wild-type *mitfa* (WT) or the mutant form (*w2*) was injected into *Tg(sox10(7.2):gfp)* (7.2) and *Tg(sox10(4.9):gfp)* (4.9) embryos. Note that at 6 hpf, only the former, but not the latter, show GFP induction. As a control, sibling embryos injected with the same constructs were fixed and examined for induction of *sox10*; note that embryos injected with the wild-type *mitfa* showed robust induction of *sox10* expression. Scale bar, 500 µm. For quantification, see Table S1.
(TIF)

Figure S5 Hdac-dependent derepression of *sox10* expression is not seen in *mitfa* mutant embryos. A–F) In situ hybridisation with *sox10* probe showing similar levels of *sox10* expression in premigratory (arrow, C) and migrating (arrowhead, C) neural crest cells of *mitfa* mutants whether treated with 1 µM Trichostatin A from 24–48 hpf (B,D,F) or in stage-matched 36 hpf DMSO control *mitfa* mutants (A,C,E). Compare effect in WT embryos shown in Figure 8. Scale bar: 100 µm.
(TIF)

Figure S6 Exploration of parameter value dependency in Model A. Concentrations (nM) of Mitfa (black) at steady state, of Sox10 (Red) at steady state, and of maximal expression of Sox10 (Blue) during relaxation, as functions of activation of Sox10 by Factor A (α), activation of Mitfa by Sox10 (γ) and repression of Sox10 by Mitfa (β). Here α = α_0_/α_1_, γ = γ_0_/γ_1_ and β = β_0_/β_1_ represent binding affinities, varied over a range of two orders of magnitude. The difficulty of realizing a state of high Mitfa expression at steady state, low steady state expression of Sox10, preceded by an appreciably different Sox10 maximal value, leads to rejection of Model A.
(TIF)

Figure S7 Exploration of parameter value dependency in Model B. Concentrations (nM) of Mitfa (black) at steady state, of Sox10 (Red) at steady state, and of maximal expression of Sox10 (Blue) during relaxation, as pair-wise functions of the affinities tuning the Hdac1-mediated repression of Mitfa activation of Sox10 (ξ), and the other regulatory interactions present in the Mitfa-Sox10 module. Here α, β, δ, θ, γ represent activation of Sox10 by Factor A, repression of Sox10 by Mitfa, activation of Mitfa by Factor Y, activation of Hdac1 by Mitfa, and activation of Mitfa by Sox10, respectively. As in Model A, Model B does not readily allow for parameter combinations giving high values of Mitfa steady state concentration, low values of Sox10 at steady state, and a substantially elevated maximum of Sox10 during relaxation.
(TIF)

Figure S8 Exploration of parameter value dependency in Model C. Concentrations (nM) of Mitfa (black) at steady state, of Sox10 (Red) at steady state, and of maximal expression of Sox10 (Blue) during relaxation, as pair-wise functions of the affinities tuning the Hdac1-mediated repression of Factor A activation of Sox10 (φ), and the other regulatory interactions present in the Mitfa-Sox10 module. Here α, β, δ, θ, γ ξ represent activation of Sox10 by Factor A, repression of Sox10 by Mitfa, activation of Factor Y by Mitfa, activation of Hdac1 by Mitfa, activation of Mitfa by Sox10, and Hdac1 repression of Mitfa activation of Sox10, respectively. In contrast to Models A and B, Model C satisfies the requirements of a high expression of Mitfa at steady state, low expression of Sox10 at steady state, and pronounced Sox10 maximum at intermediate times, over an extensive region of the parameter space.
(TIF)

Table S1 Expression of GFP or endogenous *sox10* after injection of embryos from *Tg(-7.2sox10:GFP)* and *Tg(-4.9sox10:GFP)* cross. Embryos injected with *mitfa* or *mitfa(w2)* RNA were scored for expression of sox10:GFP transgene by live observation of GFP fluorescence or for endogenous *sox10* by in situ hybridisation, and expressed as a fraction of the total number of embryos examined.
(DOC)

Text S1 Detailed description of mathematical models.
(PDF)
