# Supplementary figures and images for: An Iterative Genetic and Dynamical Modelling Approach Identifies Novel Features of the Gene Regulatory Network Underlying Melanocyte Development
Source: PLoS Genet. 2011 Sep 1;7(9):e1002265. doi: 10.1371/journal.pgen.1002265 (PMC3164703; doi:10.1371/journal.pgen.1002265)

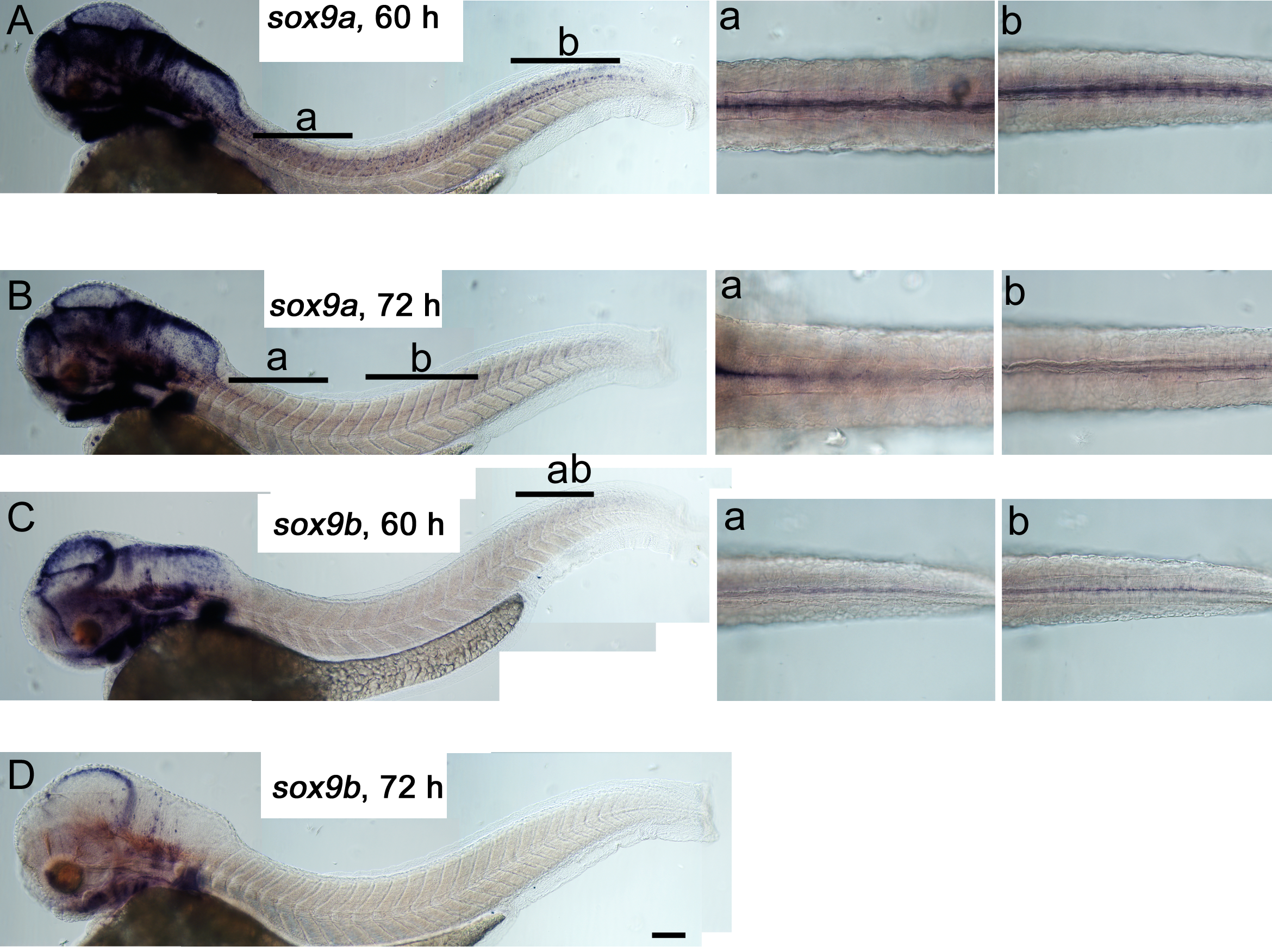

Supplement: Figure S1 — Neither sox9a nor sox9b are expressed in differentiated melanocytes. Lateral views of whole embryos (left) and dorsal views of dorsal stripe region (insets right, location indicated by lettered bars) show 60 hpf (A,C) and 72 hpf (B, D) embryos. In C, inset b shows a deeper focal plane than that in inset a. Embryos were treated with PTU to allow detection of even very weak signals. Scale bar 100 µm. (TIF) [file pgen.1002265.s001.tif]

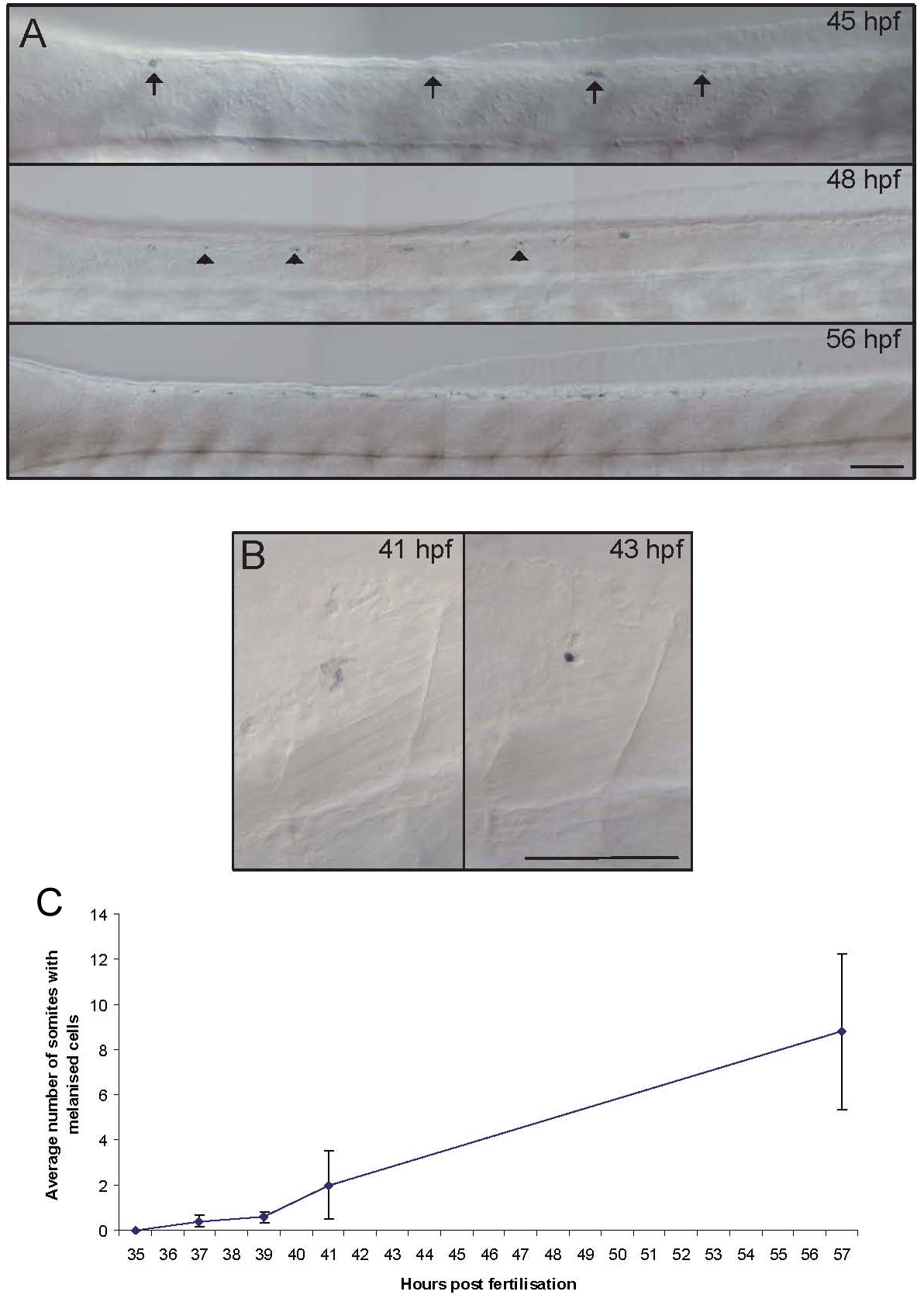

Supplement: Figure S2 — Residual melanised cells in sox10 mutants appear late and then increase with time. A) Photographs of dorsal trunk of a single embryo showing dynamic changes in residual melanised cells. Note how initially many cells show diffuse melanin (arrows) and how new melanised cells appear with time (arrowheads). B) Photographs of single melanised cell at consecutive time-points, showing change from diffuse melanin (41 hpf) to tiny, dense spot (43 hpf). C) Graphical plot of mean±s.e. number of segments containing residual melanised cells from a typical series of embryos (n = 19). (TIF) [file pgen.1002265.s002.tif]

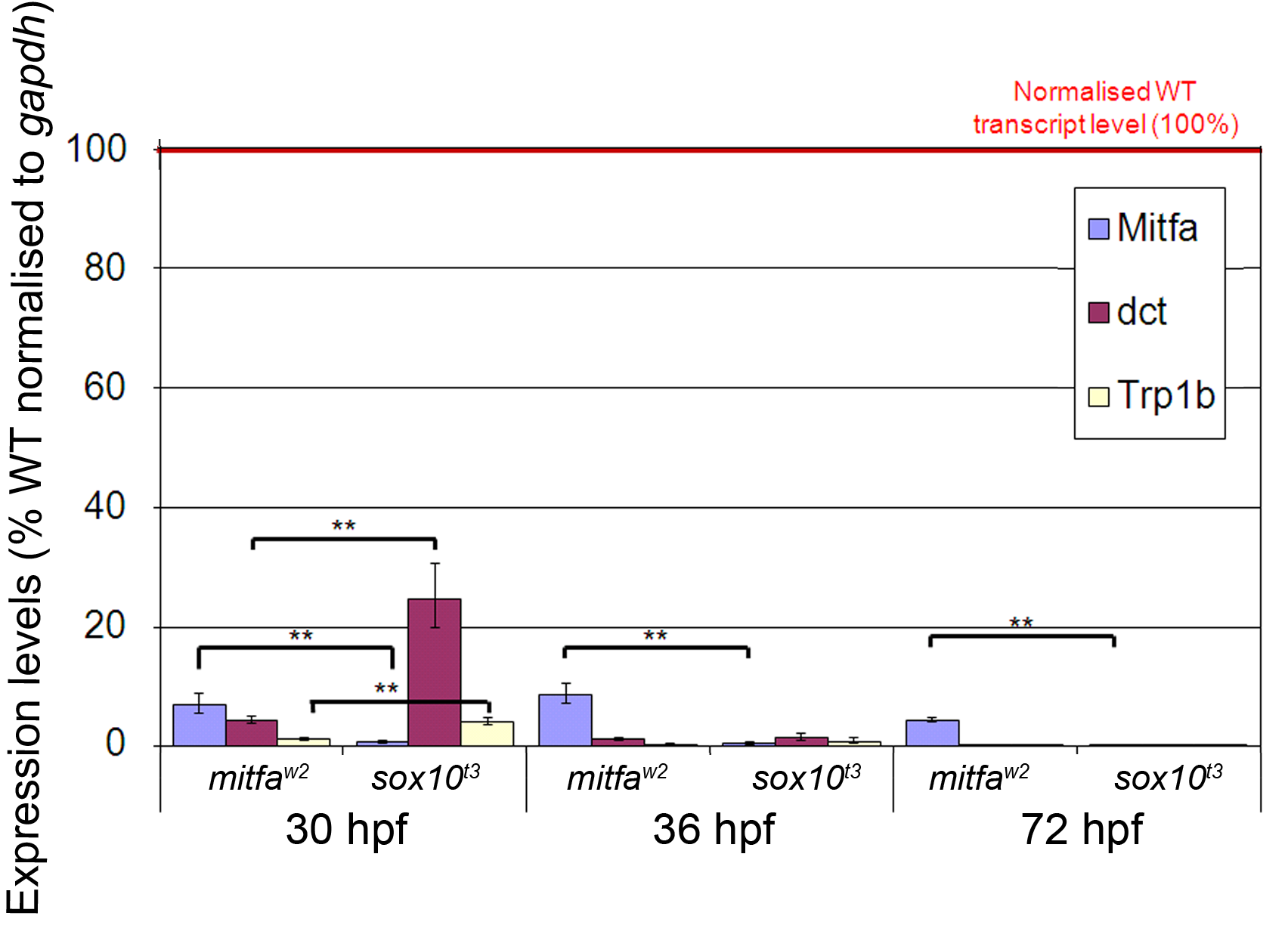

Supplement: Figure S3 — Quantitative RT-PCR of mitfa, dct and trp1b expression in wild-type (WT), mitfaw2 mutants and sox10t3 mutants. Values shown are mean±s.d. at 30 hpf, 36 hpf and 72 hpf. Expression levels in WT controls were normalised to GAPDH for each sample, and expression is shown as percentage of WT transcript expression levels normalised to GAPDH. Expression levels that were statistically significantly elevated in sox10 mutants compared with mitfa mutants are indicated (1-tailed t-test with Bonferroni correction for multiple comparisons, **). (TIF) [file pgen.1002265.s003.tif]

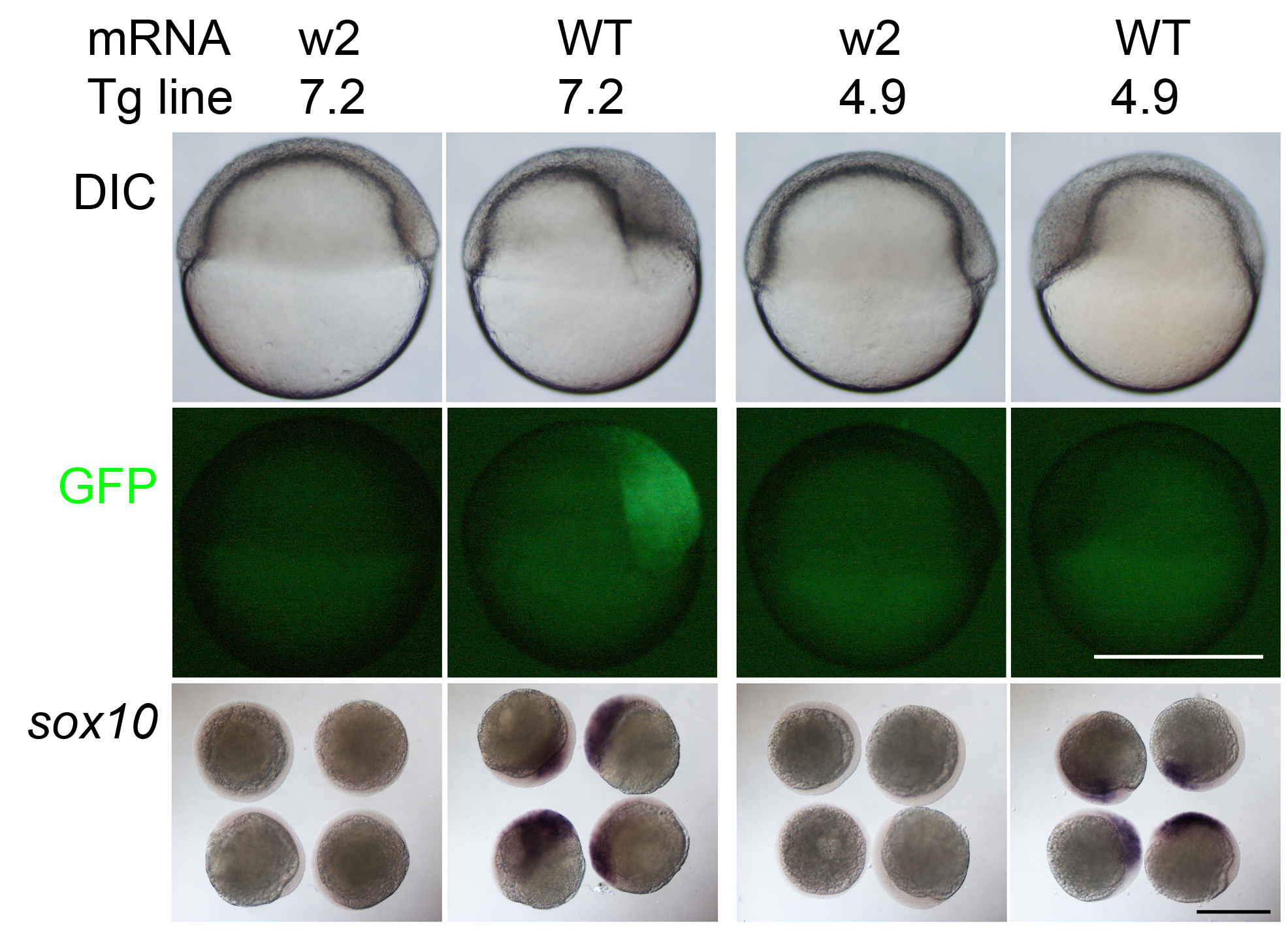

Supplement: Figure S4 — Mitfa-dependent regulation of sox10 transgenes narrows candidate regulatory elements. RNA encoding wild-type mitfa (WT) or the mutant form (w2) was injected into Tg(sox10(7.2):gfp) (7.2) and Tg(sox10(4.9):gfp) (4.9) embryos. Note that at 6 hpf, only the former, but not the latter, show GFP induction. As a control, sibling embryos injected with the same constructs were fixed and examined for induction of sox10; note that embryos injected with the wild-type mitfa showed robust induction of sox10 expression. Scale bar, 500 µm. For quantification, see Table S1. (TIF) [file pgen.1002265.s004.tif]

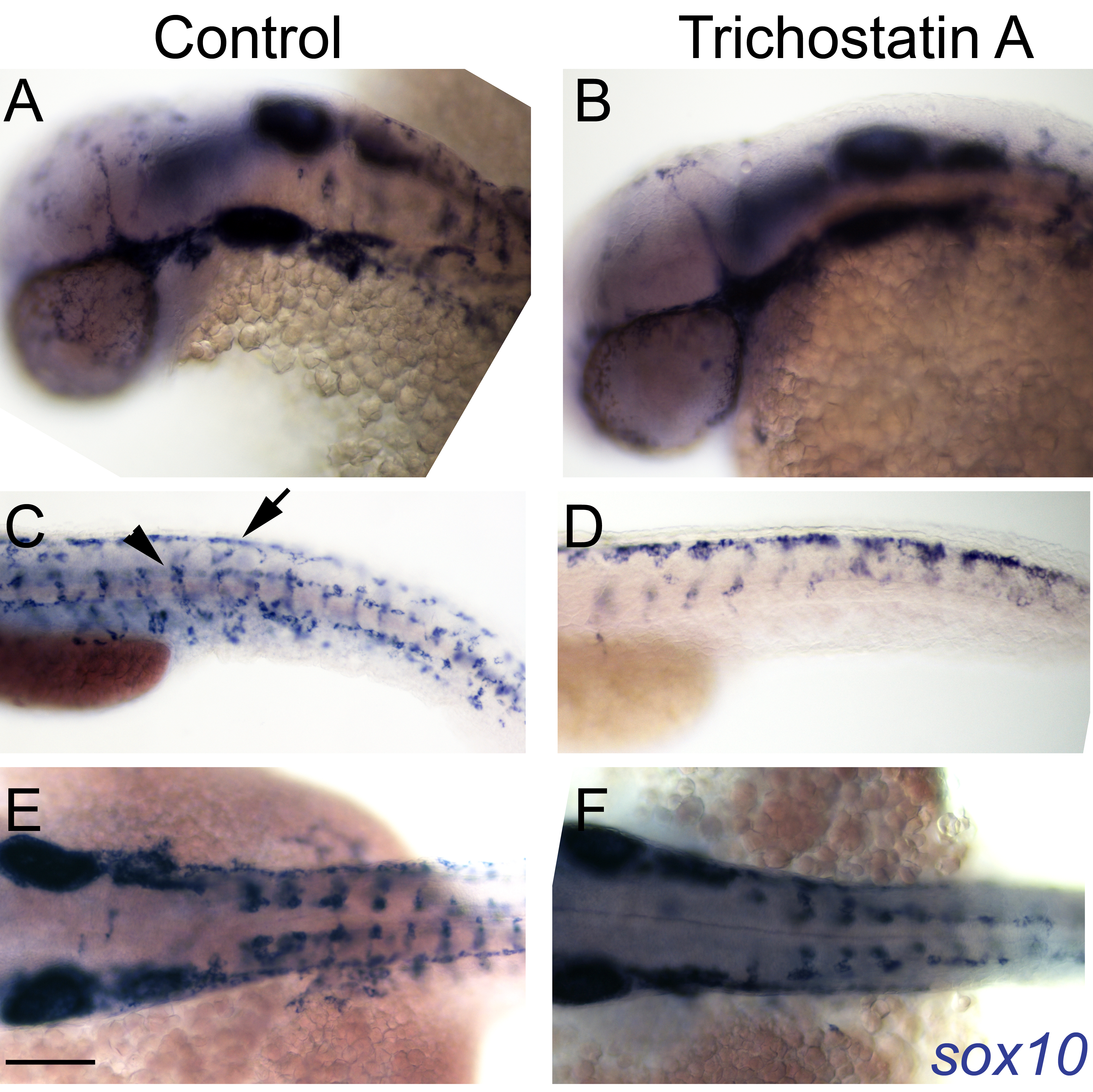

Supplement: Figure S5 — Hdac-dependent derepression of sox10 expression is not seen in mitfa mutant embryos. A–F) In situ hybridisation with sox10 probe showing similar levels of sox10 expression in premigratory (arrow, C) and migrating (arrowhead, C) neural crest cells of mitfa mutants whether treated with 1 µM Trichostatin A from 24–48 hpf (B,D,F) or in stage-matched 36 hpf DMSO control mitfa mutants (A,C,E). Compare effect in WT embryos shown in Figure 8. Scale bar: 100 µm. (TIF) [file pgen.1002265.s005.tif]

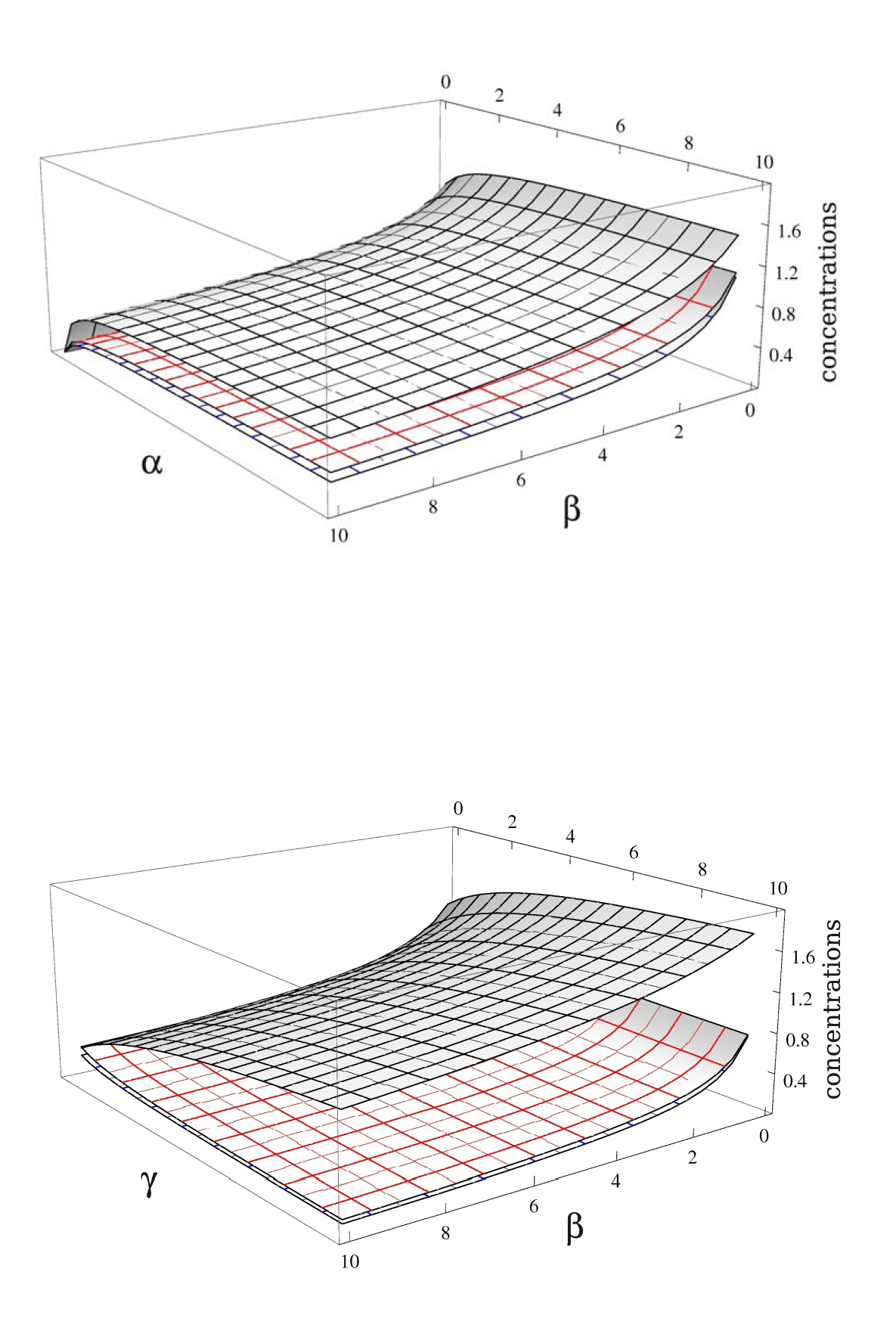

Supplement: Figure S6 — Exploration of parameter value dependency in Model A. Concentrations (nM) of Mitfa (black) at steady state, of Sox10 (Red) at steady state, and of maximal expression of Sox10 (Blue) during relaxation, as functions of activation of Sox10 by Factor A (α), activation of Mitfa by Sox10 (γ) and repression of Sox10 by Mitfa (β). Here α = α0/α1, γ = γ0/γ1 and β = β0/β1 represent binding affinities, varied over a range of two orders of magnitude. The difficulty of realizing a state of high Mitfa expression at steady state, low steady state expression of Sox10, preceded by an appreciably different Sox10 maximal value, leads to rejection of Model A. (TIF) [file pgen.1002265.s006.tif]

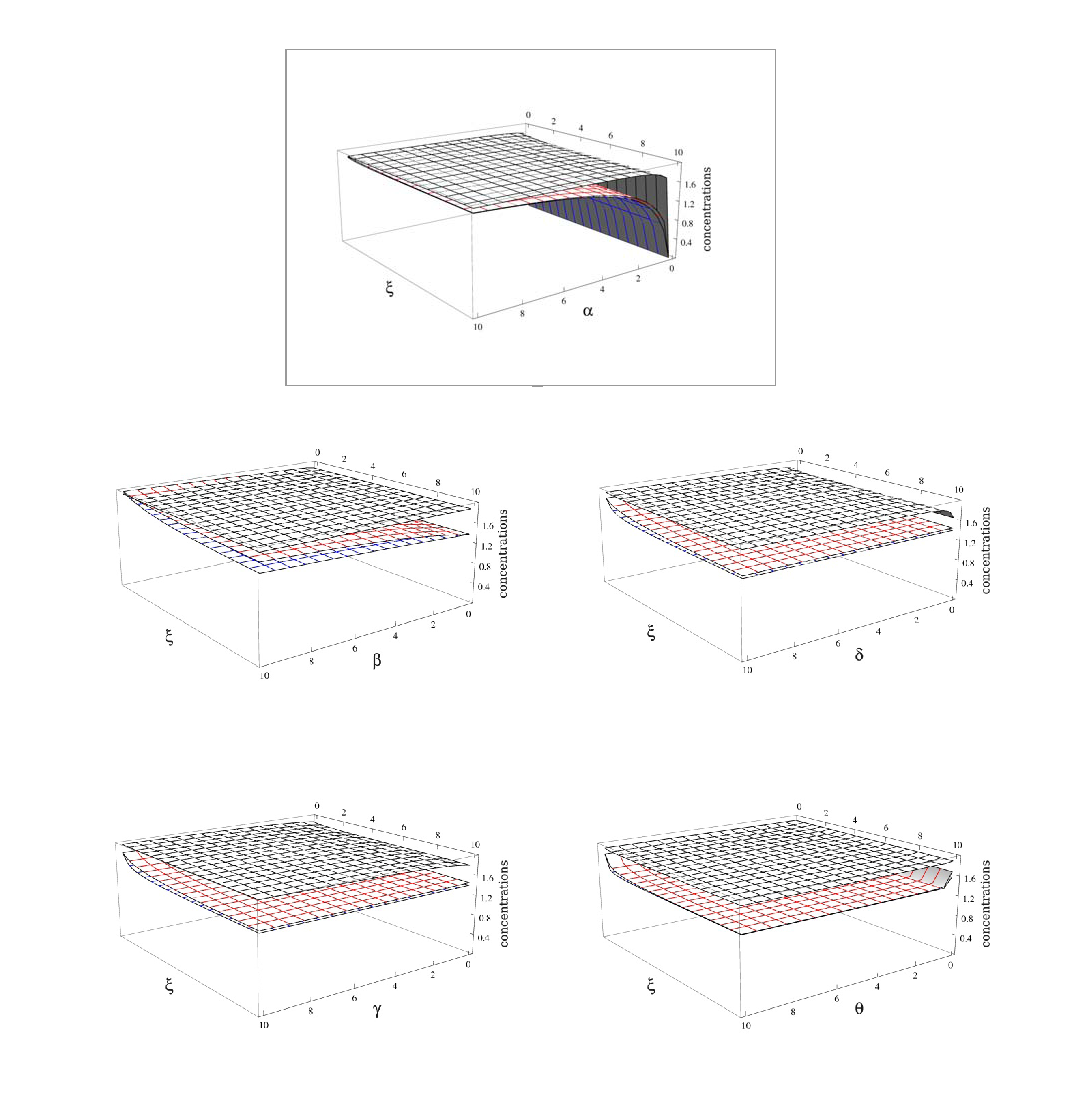

Supplement: Figure S7 — Exploration of parameter value dependency in Model B. Concentrations (nM) of Mitfa (black) at steady state, of Sox10 (Red) at steady state, and of maximal expression of Sox10 (Blue) during relaxation, as pair-wise functions of the affinities tuning the Hdac1-mediated repression of Mitfa activation of Sox10 (ξ), and the other regulatory interactions present in the Mitfa-Sox10 module. Here α, β, δ, θ, γ represent activation of Sox10 by Factor A, repression of Sox10 by Mitfa, activation of Mitfa by Factor Y, activation of Hdac1 by Mitfa, and activation of Mitfa by Sox10, respectively. As in Model A, Model B does not readily allow for parameter combinations giving high values of Mitfa steady state concentration, low values of Sox10 at steady state, and a substantially elevated maximum of Sox10 during relaxation. (TIF) [file pgen.1002265.s007.tif]

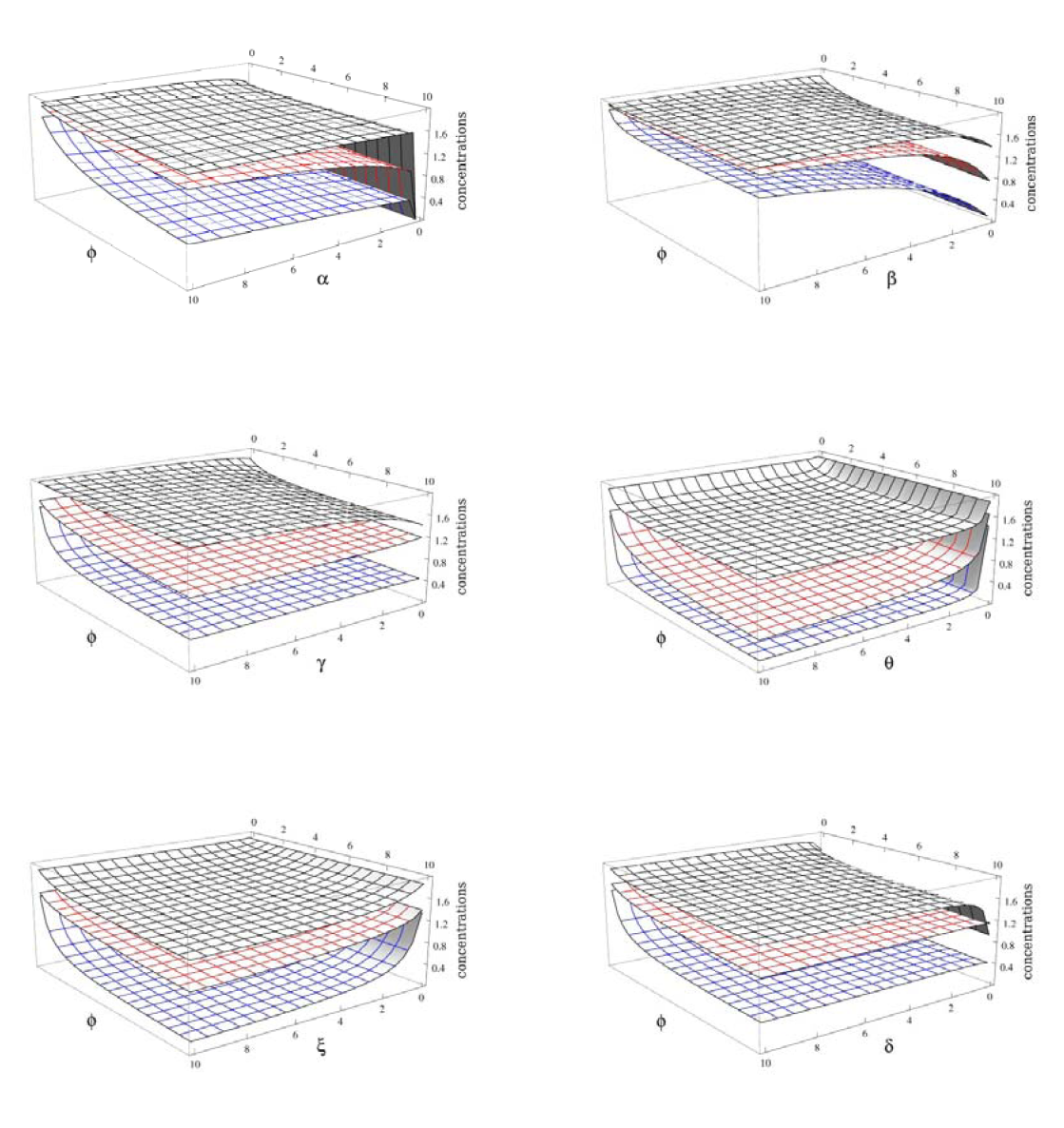

Supplement: Figure S8 — Exploration of parameter value dependency in Model C. Concentrations (nM) of Mitfa (black) at steady state, of Sox10 (Red) at steady state, and of maximal expression of Sox10 (Blue) during relaxation, as pair-wise functions of the affinities tuning the Hdac1-mediated repression of Factor A activation of Sox10 (φ), and the other regulatory interactions present in the Mitfa-Sox10 module. Here α, β, δ, θ, γ ξ represent activation of Sox10 by Factor A, repression of Sox10 by Mitfa, activation of Factor Y by Mitfa, activation of Hdac1 by Mitfa, activation of Mitfa by Sox10, and Hdac1 repression of Mitfa activation of Sox10, respectively. In contrast to Models A and B, Model C satisfies the requirements of a high expression of Mitfa at steady state, low expression of Sox10 at steady state, and pronounced Sox10 maximum at intermediate times, over an extensive region of the parameter space. (TIF) [file pgen.1002265.s008.tif]
