# Supplementary material for: An Iterative Genetic and Dynamical Modelling Approach Identifies Novel Features of the Gene Regulatory Network Underlying Melanocyte Development
Source: PLoS Genet. 2011 Sep 1;7(9):e1002265. doi: 10.1371/journal.pgen.1002265 (PMC3164703; doi:10.1371/journal.pgen.1002265)
Supplement: Table S1 — Expression of GFP or endogenous sox10 after injection of embryos from Tg(-7.2sox10:GFP) and Tg(-4.9sox10:GFP) cross. Embryos injected with mitfa or mitfa(w2) RNA were scored for expression of sox10:GFP transgene by live observation of GFP fluorescence or for endogenous sox10 by in situ hybridisation, and expressed as a fraction of the total number of embryos examined. (DOC) [file pgen.1002265.s009.doc]

**Table S1** Expression of GFP or endogenous *sox10* after injection of embryos from *Tg(-7.2sox10:GFP)* and *Tg(-4.9sox10:GFP)* cross

|  | *Tg(-7.2sox10:GFP)* | | *Tg(-4.9sox10:GFP)* | |
| --- | --- | --- | --- | --- |
| *RNA* | GFP | *sox10* | GFP | *sox10* |
| *mitfa* | 23/70 (33%) | 46/66 (70%) | 0/95 (0%) | 76/88 (86%) |
| *mitfa(w2)* | 0/59 (0%) | 0/57 (0%) | 0/103 (0%) | 0/94 (0%) |

Embryos injected with *mitfa* or *mitfa(w2)* RNA were scored for expression of sox10:GFP transgene by live observation of GFP fluorescence or for endogenous *sox10* by in situ hybridisation, and expressed as a fraction of the total number of embryos examined.
