# Supplementary material for: An Iterative Genetic and Dynamical Modelling Approach Identifies Novel Features of the Gene Regulatory Network Underlying Melanocyte Development
Source: PLoS Genet. 2011 Sep 1;7(9):e1002265. doi: 10.1371/journal.pgen.1002265 (PMC3164703; doi:10.1371/journal.pgen.1002265)
Supplement: Text S1 — Detailed description of mathematical models. (PDF) [file pgen.1002265.s010.pdf]

Supplementary Material

An Iterative Genetic and Dynamical Modelling Approach Identifies Novel Features of the Gene Regulatory Network Underlying Zebrafish Melanocyte Development

Emma R. Greenhill, Andrea Rocco, Laura Vibert, Masataka Nikaido, and Robert N. Kelsh

1. REGULATION BY ACTIVATORY TRANSCRIPTION FACTORS

Let us consider the Transcription Factor (TF) A, which regulates the two copies of gene $\mathcal{G}$, $\mathcal{G}_1$ and $\mathcal{G}_2$, one on each chromosome. Regulation proceeds by binding of A to the appropriate regulatory elements upstream of $\mathcal{G}_1$ and/or $\mathcal{G}_2$. General TF kinetics, transcription itself , mRNA export and translation are not explicitly modelled in this approach.

Upon binding of A, the regulatory element makes a transition from the "empty" state into the "occupied" (or "regulated") state, that is

$$A + e_{Ai} \underset{\alpha_1}{\overset{\alpha_0}{\rightleftharpoons}} r_{Ai} \qquad\qquad i = 1, 2 \tag{1}$$

Let $[A]$ be the concentration of A, defined as the average copy number of A per cell. Similarly we define as $[e_{Ai}]$ and $[r_{Ai}]$ the average number of empty and occupied regulatory elements respectively. Averages are taken over a population of identical cells. The constants α_0 and α_1 are the rate constants respectively for binding and unbinding of the regulatory TF. The index i refers to the two copies of regulatory elements, one for each chromosome.

Also, we define as $[G_i]$ the average number of inactive genes per cell, and as $[G_i^*]$ the average number of active genes per cell, i indicating again the two copies of the same gene, one for each chromosome. Hence $[r_{Ai}]$ is also equal to the average number of active genes $[G_i^*]$. Similarly $[e_{Ai}]$ equals the average number of inactive genes $[G_i]$.

Notice that because of the adopted definition of concentrations the probability $p(G_i^*)$ that gene $\mathcal{G}_i$ is in the active state equals the concentration $[G_i^*]$. Similarly $p(A_i) = [r_{Ai}]$ and $p(\sim A_i) = [e_{Ai}]$, where $p(A_i)$ (respectively $p(\sim A_i)$) is the probability that A is bound (respectively unbound) to its binding site.

When the gene $\mathcal{G}_i$ gets into the active state, it synthesizes the protein P with a rate g. When mRNA levels are not explicitly modelled, the rate g can be thought of as an effective rate which corresponds to the maximal expression level of each of the genes $\mathcal{G}_i$, and incorporates binding of general TFs, as well as mRNA dynamics [1–3]:

$$G_1^* \xrightarrow{\;g\;} P; \qquad\qquad G_2^* \xrightarrow{\;g\;} P. \tag{2}$$

Degradation of proteins

$$P \xrightarrow{\;d_P\;} \phi \tag{3}$$

is also implemented, d_P being the degradation rate.

The equations representing processes (1), (2), and (3) read as follows:

$$\frac{d[r_{Ai}]}{dt} = \alpha_0[A](1 - [r_{Ai}]) - \alpha_1[r_{Ai}] \qquad\qquad i = 1, 2 \tag{4}$$

$$\frac{d[P_i]}{dt} = g[r_{Ai}] - d_P[P_i] \qquad\qquad i = 1, 2 \tag{5}$$

where we used $[e_{Ai}] + [r_{Ai}] = 1$ and $[G_i^*] = [r_{Ai}]$, for $i = 1, 2$. Here $[P_i]$ is the contribution to the total concentration of protein P from the activation of gene $\mathcal{G}_i$, with $[P] = [P_1] + [P_2]$.

Let us assume that binding and unbinding of A to the regulatory element is a much faster process than protein production and degradation:

$$\alpha_0, \alpha_1 \gg g, d_P. \tag{6}$$

Then the equilibration of r_{Ai} will be faster than the equilibration of P, and we can solve equations (4) and (5) with the quasi steady state assumption $d[r_{Ai}]/dt = 0$:

$$\alpha_0[A](1-[r_{Ai}]) - \alpha_1[r_{Ai}] \quad \Rightarrow \quad [r_{Ai}] = \frac{\alpha_0[A]}{\alpha_1+\alpha_0[A]}, \tag{7}$$

whence:

$$\frac{d[P_i]}{dt} = g\frac{\alpha_0[A]}{\alpha_1+\alpha_0[A]} - d_P[P_i] \qquad\qquad i=1,2. \tag{8}$$

Summing over i we finally obtain:

$$\frac{d[P]}{dt} = g_P\frac{\alpha_0[A]}{\alpha_1+\alpha_0[A]} - d_P[P]. \tag{9}$$

where we set $g_P = 2g$.

The dynamics is driven by a function of Michaelis-Menten type, or more generally a Hill function with Hill coefficient equal to 1. This is appropriate if the TF binds to DNA as a monomer. Notice the two limits for A very small and very large: A small implies that the production rate goes to zero (no TF activating the gene), while A large reproduces the maximal expression level of the gene. Since we do not model polymerase dynamics, in particular translocation, the present approach is valid in the case of weak activators, or otherwise A small [3].

2. COOPERATIVE ACTIVATION

Complex formation

Let us assume that the efficiency of the regulatory TF is enhanced by complex formation. In other words, let us assume that one or more molecules of A first bind together to form a regulatory complex, and then this complex binds to DNA onto a single binding site. Process (1) is then modified as follows

$$A_n + e_{A_n} \underset{\alpha_1}{\overset{\alpha_0}{\rightleftharpoons}} r_{A_n} \qquad i.e. \qquad \frac{d[r_{A_n}]}{dt} \;=\; \alpha_0[A_n](1-[r_{A_n}]) - \alpha_1[r_{A_n}] \tag{10}$$

The symbol A_n represents the complex of n monomers of A. The corresponding reaction reads:

$$nA \underset{k_2}{\overset{k_1}{\rightleftharpoons}} A_n \tag{11}$$

and accordingly

$$\frac{d[A_n]}{dt} = k_1[A]^n - k_2[A_n] \tag{12}$$

We assume again time scale separation among the different processes involved, namely:

$$k_1, k_2 \gg \alpha_0, \alpha_1 \gg g_P, d_P. \tag{13}$$

In this way, the association reaction equilibrates first, and we can safely replace $[A_n] = (k_1/k_2)[A]^n$ in equation (10). The same procedure used in the previous section then gives:

$$\frac{d[P]}{dt} = g_P\frac{\frac{\alpha_0 k_1}{k_2}[A]^n}{\alpha_1+\frac{\alpha_0 k_1}{k_2}[A]^n} - d_P[P]. \tag{14}$$

The production rate results in the well-known Hill function. The larger n, the more sigmoidal the shape of the Hill function.

Two binding sites: The AND gate

Let us consider now the case when two different activators A and B bind independently to two different regulatory elements. By AND gate, we mean the situation when binding of both A and B is needed in order for gene $\mathcal{G}$ to be activated.

The processes involved are:

$$A + e_A \underset{\alpha_1}{\overset{\alpha_0}{\rightleftharpoons}} r_A \qquad i.e. \qquad \frac{d[r_A]}{dt} = \alpha_0[A](1-[r_A]) - \alpha_1[r_A], \tag{15}$$

$$B + e_B \underset{\beta_1}{\overset{\beta_0}{\rightleftharpoons}} r_B \qquad i.e. \qquad \frac{d[r_B]}{dt} = \beta_0[B](1-[r_B]) - \beta_1[r_B], \tag{16}$$

which at steady state give

$$[r_A] = \frac{\alpha_0[A]}{\alpha_1 + \alpha_0[A]} \qquad \text{and} \qquad [r_B] = \frac{\beta_0[B]}{\beta_1 + \beta_0[B]}. \tag{17}$$

The average number of activated genes is

$$[G^*] = p(G^*) = p(A,B) = p(A)p(B) = [r_A][r_B]. \tag{18}$$

where $p(A,B)$ is the joint probability that both A and B are bound. Therefore:

$$\frac{d[P]}{dt} = g_P \frac{\alpha_0[A]}{\alpha_1 + \alpha_0[A]} \cdot \frac{\beta_0[B]}{\beta_1 + \beta_0[B]} - d_P[P]. \tag{19}$$

The generalization of more than two TF's binding each to its specific binding site is straightforward.

Two binding sites: The OR gate

In this case gene $\mathcal{G}$ is activated by the binding of at least one of either A or B. Activation of $\mathcal{G}$ implies

$$[G^*] = p(G^*) = p(A, \sim B) + p(\sim A, B) + p(A,B) = [r_A](1-[r_B]) + (1-[r_A])[r_B] + [r_A][r_B], \tag{20}$$

where $[r_A]$ and $[r_B]$ are as given by equations (17). This leads to the following equation for protein production:

$$\frac{d[P]}{dt} = g_P \frac{\alpha_0\beta_1[A] + \beta_0\alpha_1[B] + \alpha_0\beta_0[A][B]}{(\alpha_1 + \alpha_0[A])(\beta_1 + \beta_0[B])} - d_P[P]. \tag{21}$$

Notice that the maximal expression rate g_P is reached in the limit $[A] \to \infty$, or $[B] \to \infty$, or both. Only for both $[A] \to 0$ and $[B] \to 0$, expression drops to 0.

3. COMPETITIVE ACTIVATION

Consider now two activators A and B competing for binding to the same binding site. In order to calculate the concentration of active genes, we proceed as follows:

$$[G^*] = p(G^*) = p(A, \sim B) + p(\sim A, B) \tag{22}$$

with

$$p(A, \sim B) = p(A) = p(A| \sim B)p(\sim B) = p(A| \sim B)(1 - p(B)), \tag{23}$$

$$p(\sim A, B) = p(\sim A, |B)p(B) = p(B) \tag{24}$$

where we used Bayes' Theorem, and the fact that binding of A and binding of B are mutually exclusive events. Notice that the conditional probabilities $p(A| \sim B)$ and $p(B| \sim A)$ correspond to $[r_A]$ and $[r_B]$ respectively:

$$p(A| \sim B) = [r_A] = \frac{\alpha_0[A]}{\alpha_1 + \alpha_0[A]} \tag{25}$$

$$p(B| \sim A) = [r_B] = \frac{\beta_0[B]}{\beta_1 + \beta_0[B]} \tag{26}$$

Also:

$$p(B) = p(B, \sim A) = p(B|\sim A)p(\sim A) = p(B|\sim A)(1 - p(A)) \tag{27}$$

Combining (23) and (27), we obtain:

$$p(A) = \frac{[r_A] - [r_A][r_B]}{1 - [r_A][r_B]} \qquad \text{and} \qquad p(B) = \frac{[r_B] - [r_A][r_B]}{1 - [r_A][r_B]} \tag{28}$$

and finally

$$\frac{d[P]}{dt} = g_P \frac{\alpha_0\beta_1[A] + \beta_0\alpha_1[B]}{\alpha_1\beta_1 + \alpha_1\beta_0[B] + \alpha_0\beta_1[A]} - d_P[P]. \tag{29}$$

4. REGULATION BY REPRESSIVE TRANSCRIPTION FACTORS

Let us consider gene $\mathcal{G}$ in the active state G^*. When a repressor R binds to the proper binding site it downregulates expression of $\mathcal{G}$. The dynamics is the same as the dynamics for activation with the only difference that the active state G^* is the one with the repressor unbound.

Therefore

$$\frac{d[r_R]}{dt} = \alpha_0[R](1 - [r_R]) - \alpha_1[r_R] \tag{30}$$

$$\frac{d[P]}{dt} = g_P(1 - [r_R]) - d_P[P], \tag{31}$$

where we used $[G^*] = p(G^*) = p(\sim R) = 1 - p(R) = 1 - [r_R]$. The quasi steady state solution reads:

$$\frac{d[P]}{dt} = g_P \frac{\alpha_1}{\alpha_1 + \alpha_0[R]} - d_P[P]. \tag{32}$$

where again we assumed $\alpha_0, \alpha_1 \gg g_P, d_P$. The expression level of the gene is now maximal when the concentration of repressor is zero. On the other hand, for large repressor concentrations the protein production drops to zero.

5. NON-COMPETITIVE REPRESSION

Let us consider now a regulatory process that involves an activator and a repressor acting on two different binding sites. This case is formally very similar to activation through two activatory proteins as discussed in section 3, the only difference being the computation of the number of active genes.

The relevant processes read:

$$A + e_A \underset{\alpha_1}{\overset{\alpha_0}{\rightleftharpoons}} r_A \qquad \text{and} \qquad R + e_R \underset{\beta_1}{\overset{\beta_0}{\rightleftharpoons}} r_R \tag{33}$$

with the concentrations $[r_A]$ and $[r_R]$ at steady state given by:

$$[r_A] = \frac{\alpha_0[A]}{\alpha_1 + \alpha_0[A]} \qquad \text{and} \qquad [r_R] = \frac{\beta_0[R]}{\beta_1 + \beta_0[R]}. \tag{34}$$

Since now activation depends upon the regulatory element for the repressor to stay unbound, we have now $[G^*] = p(G^*) = p(A, \sim R) = p(A)(1 - p(R)) = [r_A](1 - [r_B])$, and therefore:

$$\frac{d[P]}{dt} = g_P \frac{\alpha_0[A]}{\alpha_1 + \alpha_0[A]} \cdot \frac{\beta_1}{\beta_1 + \beta_0[R]} - d_P[P]. \tag{35}$$

6. REGULATION BY COMPETITIVE ACTIVATION AND REPRESSION

Consider now an activator A and a repressor R competing for binding to the same binding site. The concentration of active genes reads:

$$[G^*] = p(G^*) = p(A, \sim R) = p(A| \sim R)p(\sim R) = p(A| \sim R)(1 - p(R)). \tag{36}$$

By using (28) this turns out to be

$$[G^*] = \frac{[r_A] - [r_A][r_R]}{1 - [r_A][r_R]}, \tag{37}$$

and finally

$$\frac{d[P]}{dt} = g_P \frac{\alpha_0\beta_1[A]}{\alpha_1\beta_1 + \alpha_0\beta_1[A] + \alpha_1\beta_0[R]} - d_P[P]. \tag{38}$$

7. MELANOCYTE DIFFERENTIATION IN ZEBRAFISH – MODEL A

Let us consider the gene regulatory network depicted in Fig. 9, Model A. The transcription factor A is provided externally so as to activate *Sox10*. The gene X is an unknown putative gene, which plays the same role as Phox2A in the GRN for sympathetic neurons [4]. For simplicity of notation, we define S to be the concentration of *Sox10* and M the concentration of *Mitfa*. We make the following assumptions:

- *Sox10* is activated by a complex regulatory process which involves a number of transcription factors. We model this as an effective TF, which binds as a monomer to the appropriate binding site, and for which we assume the following step-like time dependency:

$$A(t) = \frac{A_0}{1 + \exp\left(\beta(t_A - t)\right)}. \tag{39}$$

  Parameter A_0 fixes the maximum value of A, β fixes the rising time of the A signal, and t_A defines the position in time of the step.

- Binding of *Sox10* occurs in both monomer and dimer forms [5]. For simplicity we assume that only one binding site is present, and *Sox10* monomers and dimers can bind in a competitive fashion.
- All other proteins produced in the circuit, acting as regulatory TF's, bind as monomers to the respective regulatory elements.
- Activators and repressors of the same gene bind non-competitively at different binding sites.
- Binding and unbinding of TFs is fast, so that a quasi steady state approximation is valid.

The regulatory processes and respective equations read:

$$S: \begin{cases} A + e_A^{(S)} \underset{\alpha_1}{\overset{\alpha_0}{\rightleftharpoons}} r_A^{(S)} & i.e. \quad \dfrac{dr_A^{(S)}}{dt} = \alpha_0 A(1 - r_A^{(S)}) - \alpha_1 r_A^{(S)} \\ M + e_M^{(S)} \underset{\beta_1}{\overset{\beta_0}{\rightleftharpoons}} r_M^{(S)} & i.e. \quad \dfrac{dr_M^{(S)}}{dt} = \beta_0 M(1 - r_M^{(S)}) - \beta_1 r_M^{(S)} \end{cases} \tag{40}$$

$$M: \begin{cases} S + e_S^{(M)} \underset{\gamma_1^{(1)}}{\overset{\gamma_0^{(1)}}{\rightleftharpoons}} r_S^{(M)} & i.e. \quad \dfrac{dr_S^{(M)}}{dt} = \gamma_0^{(1)} S(1 - r_S^{(M)} - r_{S_2}^{(M)}) - \gamma_1^{(1)} r_S^{(M)} \\ S + S \underset{k_2}{\overset{k_1}{\rightleftharpoons}} S_2 & i.e. \quad \dfrac{dS_2}{dt} = k_1 S^2 - k_2 S_2 \\ S_2 + e_S^{(M)} \underset{\gamma_1^{(2)}}{\overset{\gamma_0^{(2)}}{\rightleftharpoons}} r_{S_2}^{(M)} & i.e. \quad \dfrac{dr_{S_2}^{(M)}}{dt} = \gamma_0^{(2)} S_2(1 - r_S^{(M)} - r_{S_2}^{(M)}) - \gamma_1^{(2)} r_{S_2}^{(M)} \end{cases} \tag{41}$$

$$X: \quad \begin{cases} M + e_M^{(X)} \underset{\eta_1}{\overset{\eta_0}{\rightleftharpoons}} r_M^{(X)} & i.e. \quad \dfrac{dr_M^{(X)}}{dt} = \eta_0 M(1 - r_M^{(X)}) - \eta_1 r_M^{(X)} \\ S + e_S^{(X)} \underset{\zeta_1}{\overset{\zeta_0}{\rightleftharpoons}} r_S^{(X)} & i.e. \quad \dfrac{dr_S^{(X)}}{dt} = \zeta_0 S(1 - r_S^{(X)}) - \zeta_1 r_S^{(X)} \end{cases}, \tag{42}$$

where by $e_F^{(G)}$ and $r_F^{(G)}$ we indicate the average number of empty (respectively occupied) binding sites for TF F in the promoter region of gene G.

Let us consider now the rate equation for S:

$$\begin{aligned} \frac{dS}{dt} &= g_S r_A^{(S)}(1 - r_M^{(S)}) - d_S S \\ &-\gamma_0^{(1)} S(1 - r_S^{(M)} - r_{S_2}^{(M)}) + \gamma_1^{(1)} r_S^{(M)} - k_1 S^2 + k_2 S_2 - \zeta_0 S(1 - r_S^{(X)}) + \zeta_1 r_S^{(X)} \end{aligned} \tag{43}$$

The quasi steady state approximation means to set to zero the derivatives in equations (40), (41), and (42), but to keep the derivative in (43), and accordingly to get rid of the terms in the second line of eq. (43). Therefore (43) becomes:

$$\frac{dS}{dt} = g_S r_A^{(S)}(1 - r_M^{(S)}) - d_S S = g_S \frac{\alpha_0 A}{\alpha_1 + \alpha_0 A} \cdot \frac{\beta_1}{\beta_1 + \beta_0 M} - d_S S. \tag{44}$$

Similarly, equations for M and X read:

$$\frac{dM}{dt} = g_M \frac{\gamma_0^{(1)}\gamma_1^{(2)} S + (\gamma_0^{(2)}\gamma_1^{(1)} k_1/k_2) S^2}{\gamma_1^{(1)}\gamma_1^{(2)} + \gamma_1^{(2)}\gamma_0^{(1)} S + (\gamma_1^{(1)}\gamma_0^{(2)} k_1/k_2) S^2} - d_M M, \tag{45}$$

$$\frac{dX}{dt} = g_X \frac{\eta_0 M}{\eta_1 + \eta_0 M} \cdot \frac{\zeta_1}{\zeta_1 + \zeta_0 S} - d_X X, \tag{46}$$

The set of equations (44), (45), and (46) represent the full dynamics of the network depicted in Fig. 9 (Model A).

8. MELANOCYTE DIFFERENTIATION IN ZEBRAFISH – MODEL B

Let us consider the gene regulatory network depicted in Fig. 9 (Model B). Activation of *Sox10* is followed by activation of *Mitfa* and *Factor Y*, which define a positive feedback loop. Also, repression of *Mitfa* upon *Sox10* is now further detailed by introducing *Mitfa* activation of *Sox10* inhibited by *Hdac1*, and this is effectively modelled as a competitive activation/repression process (as from section 6). On top of the assumptions made already for Model A, we also assume the OR gate (see section 2) for the activation of *Mitfa* by *Sox10* and *Factor Y*.

Again for simplicity of notation, we define H as the concentration of *Hdac1*, T as the concentration of *Tyrp1*, and D as the concentration of *Dct*. Because of the topology of the network, we report equations and numerical data only for *Tyrp1* and *Dct*. The regulatory processes and respective equations then read:

$$S: \quad \begin{cases} A + e_A^{(S)} \underset{\alpha_1}{\overset{\alpha_0}{\rightleftharpoons}} r_A^{(S)} & i.e. \quad \dfrac{dr_A^{(S)}}{dt} = \alpha_0 A(1 - r_A^{(S)}) - \alpha_1 r_A^{(S)} \\ M + e_M^{(S)} \underset{\beta_1}{\overset{\beta_0}{\rightleftharpoons}} r_M^{(S)} & i.e. \quad \dfrac{dr_M^{(S)}}{dt} = \beta_0 M(1 - r_M^{(S)} - r_H^{(S)}) - \beta_1 r_M^{(S)} \\ H + e_M^{(S)} \underset{\xi_1}{\overset{\xi_0}{\rightleftharpoons}} r_H^{(S)} & i.e. \quad \dfrac{dr_H^{(S)}}{dt} = \xi_0 H(1 - r_M^{(S)} - r_H^{(S)}) - \xi_1 r_H^{(S)} \end{cases} \tag{47}$$

$$M: \begin{cases} S + e_S^{(M)} \underset{\gamma_1^{(1)}}{\overset{\gamma_0^{(1)}}{\rightleftharpoons}} r_S^{(M)} & i.e. \quad \frac{dr_S^{(M)}}{dt} = \gamma_0^{(1)} S(1 - r_S^{(M)} - r_{S_2}^{(M)}) - \gamma_1^{(1)} r_S^{(M)} \\ S + S \underset{k_2}{\overset{k_1}{\rightleftharpoons}} S_2 & i.e. \quad \frac{dS_2}{dt} = k_1 S^2 - k_2 S_2 \\ S_2 + e_S^{(M)} \underset{\gamma_1^{(2)}}{\overset{\gamma_0^{(2)}}{\rightleftharpoons}} r_{S_2}^{(M)} & i.e. \quad \frac{dr_{S_2}^{(M)}}{dt} = \gamma_0^{(2)} S_2(1 - r_S^{(M)} - r_{S_2}^{(M)}) - \gamma_1^{(2)} r_{S_2}^{(M)} \\ Y + e_Y^{(M)} \underset{\sigma_1}{\overset{\sigma_0}{\rightleftharpoons}} r_Y^{(M)} & i.e. \quad \frac{dr_Y^{(M)}}{dt} = \sigma_0 Y(1 - r_Y^{(M)}) - \sigma_1 r_Y^{(M)} \end{cases} \tag{48}$$

$$Y: \begin{cases} M + e_M^{(Y)} \underset{\delta_1}{\overset{\delta_0}{\rightleftharpoons}} r_M^{(Y)} & i.e. \quad \frac{dr_M^{(Y)}}{dt} = \delta_0 M(1 - r_M^{(Y)}) - \delta_1 r_M^{(Y)} \end{cases} \tag{49}$$

$$H: \begin{cases} M + e_M^{(H)} \underset{\vartheta_1}{\overset{\vartheta_0}{\rightleftharpoons}} r_M^{(H)} & i.e. \quad \frac{dr_M^{(H)}}{dt} = \vartheta_0 M(1 - r_M^{(H)}) - \vartheta_1 r_M^{(H)} \end{cases} \tag{50}$$

$$T: \begin{cases} M + e_M^{(T)} \underset{\mu_1}{\overset{\mu_0}{\rightleftharpoons}} r_M^{(T)} & i.e. \quad \frac{dr_M^{(T)}}{dt} = \mu_0 M(1 - r_M^{(T)}) - \mu_1 r_M^{(T)} \end{cases} \tag{51}$$

$$D: \begin{cases} M + e_M^{(D)} \underset{\lambda_1}{\overset{\lambda_0}{\rightleftharpoons}} r_M^{(D)} & i.e. \quad \frac{dr_M^{(D)}}{dt} = \lambda_0 M(1 - r_M^{(D)}) - \lambda_1 r_M^{(D)} \\ S + e_S^{(D)} \underset{\nu_1}{\overset{\nu_0}{\rightleftharpoons}} r_S^{(D)} & i.e. \quad \frac{dr_S^{(D)}}{dt} = \nu_0 S(1 - r_S^{(D)}) - \nu_1 r_S^{(D)} \end{cases} \tag{52}$$

Under the quasi steady state approximation, the relative equations read:

$$\frac{dS}{dt} = g_S[r_A(1 - r_{MH}) + (1 - r_A) r_{MH} + r_A r_{MH}] - d_S S, \tag{53}$$

$$\text{with} \quad r_A = \frac{\alpha_0 A}{\alpha_1 + \alpha_0 A} \quad \text{and} \quad r_{MH} = \frac{\beta_0 \xi_1 M}{\beta_1 \xi_1 + \beta_0 \xi_1 M + \beta_1 \xi_0 H},$$

$$\frac{dM}{dt} = g_M \frac{\left[\gamma_0^{(1)} \gamma_1^{(2)} S + (\gamma_0^{(2)} \gamma_1^{(1)} k_1/k_2) S^2\right](\sigma_1 + \sigma_0 Y) + \gamma_1^{(1)} \gamma_1^{(2)} \sigma_0 Y}{\left[\gamma_1^{(1)} \gamma_1^{(2)} + \gamma_0^{(1)} \gamma_1^{(2)} S + (\gamma_0^{(2)} \gamma_1^{(1)} k_1/k_2) S^2\right](\sigma_1 + \sigma_0 Y)} - d_M M, \tag{54}$$

$$\frac{dY}{dt} = g_Y \frac{\delta_0 M}{\delta_1 + \delta_0 M} - d_Y Y, \tag{55}$$

$$\frac{dH}{dt} = g_H \frac{\vartheta_0 M}{\vartheta_1 + \vartheta_0 M} - d_H H, \tag{56}$$

$$\frac{dT}{dt} = g_T \frac{\mu_0 M}{\mu_1 + \mu_0 M} - d_T T. \tag{57}$$

$$\frac{dD}{dt} = g_D \frac{\lambda_0 M}{\lambda_1 + \lambda_0 M} \cdot \frac{\nu_1}{\nu_1 + \nu_0 S} - d_D D. \tag{58}$$

The set of equations (53), (54), (55), (56), (57), and (58) represents the full dynamics of the network depicted in Fig. 9 (Model B). At steady state the dynamics is determined by S, M, and Y only, and can be determined by setting all derivatives to zero. In particular it is interesting to consider the *Sox10* mutant, which can be selected by imposing

$\gamma_0^{(1)} = \gamma_0^{(2)} = \nu_0 = 0$. In this case the set of equations (53)-(58) becomes

$$S = D_S[r_A(1 - r_{MH}) + (1 - r_A)r_{MH} + r_A r_{MH}] \tag{59}$$

$$M = D_M \frac{\sigma_0 Y}{\sigma_1 + \sigma_0 Y}, \tag{60}$$

$$Y = D_Y \frac{\delta_0 M}{\delta_1 + \delta_0 M}, \tag{61}$$

$$H = D_H \frac{\vartheta_0 M}{\vartheta_1 + \vartheta_0 M} \tag{62}$$

$$T = D_T \frac{\mu_0 M}{\mu_1 + \mu_0 M}, \tag{63}$$

$$D = D_D \frac{\lambda_0 M}{\lambda_1 + \lambda_0 M}, \tag{64}$$

where $D_S = g_S/d_S$, $D_M = g_M/d_M$, $D_Y = g_Y/d_Y$, $D_H = g_H/d_H$, $D_T = g_T/d_T$, $D_D = g_D/d_D$, and r_A and r_{MH} are as from (53). It is clear that in this case M and Y alone determine the full dynamics, their equations being coupled as a manifestation of the $M - Y$ feedback loop. For M and Y two steady states are present, the null one, $(M, Y) = (0, 0)$, and

$$M = \frac{D_M D_Y \delta_0 \sigma_0 - \delta_1 \sigma_1}{\delta_0(D_Y \sigma_0 + \sigma_1)}, \tag{65}$$

$$Y = \frac{D_M D_K \delta_0 \sigma_0 - \delta_1 \sigma_1}{\sigma_0(D_M \delta_0 + \delta_1)}. \tag{66}$$

For $D_M D_Y \delta_0 \sigma_0 - \delta_1 \sigma_1 > 0$ the non zero steady state is stable and the null steady state is unstable. In contrast, for $D_M D_Y \delta_0 \sigma_0 - \delta_1 \sigma_1 < 0$ the null steady state is stable, while the non zero one is unstable.

9. MELANOCYTE DIFFERENTIATION IN ZEBRAFISH – MODEL C

Model B shows three drawbacks. The first one relates to the fact that *Mitfa* repression of *Sox10* mediated by *Hdac1* does not appear sufficient to drive Sox10 expression below a detection threshold after its initial high expression phase. We cure this by imposing *Hdac1* inhibition onto *Factor A* as well, as shown in Fig. 9 (Model C).

The second drawback relates to the unstable features of the null (M, Y) steady state as from (65), (66) for $D_M D_Y \delta_0 \sigma_0 - \delta_1 \sigma_1 > 0$ in the *Sox10* mutant. In this case the basal expression of either *Mitfa* or *Factor Y* would be sufficient to start the feedback loop, and get all target genes normally expressed. This is at odds with experimental observation. To avoid this circumstance we insert a threshold mechanism based on *Mitfa* and *Factor Y* concentration, which effectively mimicks a regulatory dynamics not characterized in the present model.

The third drawback is related to the behaviour of both *Mitfa* and *Sox10* mutants. The experimentally observed existence of a transient *Dct* signal in both mutants implies the existence of a parallel pathway that provides only weak activation and is overwhelmed in the wild type situation, but becomes the predominant one in mutants. We assume this pathway to be mediated by *Factor Z*, and driven by a transient activator, *Factor B*.

We then consider the gene regulatory network depicted in Fig. 9 (Model C). With the same conventions and assumptions of the previous section, the corresponding set of equations reads:

$$\frac{dZ}{dt} = g_Z \frac{\rho_0 B}{\rho_1 + \rho_0 B} - d_Z Z, \tag{67}$$

$$\frac{dS}{dt} = g_S[r_{AH}(1 - r_{MH}) + (1 - r_{AH})r_{MH} + r_{AH} r_{MH}] - d_S S, \tag{68}$$

$$\text{with} \quad r_{AH} = \frac{\alpha_0 \varphi_1 A}{\alpha_1 \varphi_1 + \alpha_0 \varphi_1 A + \alpha_1 \varphi_0 H} \quad \text{and} \quad r_{MH} = \frac{\beta_0 \xi_1 M}{\beta_1 \xi_1 + \beta_0 \xi_1 M + \beta_1 \xi_0 H},$$

$$\frac{dM}{dt} = g_M \frac{\left[\gamma_0^{(1)} \gamma_1^{(2)} S + (\gamma_0^{(2)} \gamma_1^{(1)} k_1/k_2) S^2\right](\sigma_1 + \sigma_0 \Theta(Y - Y^*) Y) + \gamma_1^{(1)} \gamma_1^{(2)} \sigma_0 \Theta(Y - Y^*) Y}{\left[\gamma_1^{(1)} \gamma_1^{(2)} + \gamma_0^{(1)} \gamma_1^{(2)} S + (\gamma_0^{(2)} \gamma_1^{(1)} k_1/k_2) S^2\right](\sigma_1 + \sigma_0 \Theta(Y - Y^*) Y)} - d_M M, \tag{69}$$

$$\frac{dY}{dt} = g_Y \frac{\delta_0 \Theta(M - M^*) M}{\delta_1 + \delta_0 \Theta(M - M^*) M} - d_Y Y, \tag{70}$$

$$\frac{dH}{dt} = g_H \frac{\vartheta_0 M}{\vartheta_1 + \vartheta_0 M} - d_H H, \tag{71}$$

$$\frac{dT}{dt} = g_T \frac{\mu_0 M}{\mu_1 + \mu_0 M} - d_T T. \tag{72}$$

$$\frac{dD}{dt} = g_D \frac{\lambda_0 \varepsilon_1 M + \lambda_1 \varepsilon_0 Z + \lambda_0 \varepsilon_0 M Z}{(\lambda_1 + \lambda_0 M)(\varepsilon_1 + \varepsilon_0 Z)} \cdot \frac{\nu_1}{\nu_1 + \nu_0 S} - d_D D. \tag{73}$$

Here B is given by the equation

$$B(t) = \frac{B_0}{1 + \exp\left(\beta(t - t_2)\right)} - \frac{B_0}{1 + \exp\left(\beta(t - t_1)\right)}, \tag{74}$$

$\Theta(x)$ is the Heaviside function, defined as $\Theta(x) = 1$ for $x > 0$, and $\Theta(x) = 0$ otherwise, and M^* and Y^* are threshold parameters. Also, φ_0 and φ_1 are binding and unbinding rates of *Hdac1* to the promoter of *Sox10* (competitive binding to the same binding site of *Factor A*). Finally, Z refers to the concentration of *Factor Z*, ρ_0 and ρ_1 correspond to binding/unbinding rates of B to the *Factor Z* promoter, while ε_0 and ε_1 are the binding/unbinding rates of *Factor Z* to the promoter of *Dct*.

---

[1] N. Rosenfeld, M.B. Elowitz, U. Alon, J. Mol. Biol. 323, 785-793 (2002).
[2] A. Loinger and O. Biham, Phys. Rev. E 76, 051917 (2007).
[3] S. B.-T. de-Leon, E.H. Davidson, Dev. Biol.325, 317-328 (2009).
[4] J. Kim, L. Lo, E. Dormand, D.J. Anderson, Neuron 38, 17-31 (2003).
[5] A. Ludwig, S. Rehberg, M. Wegner, FEBS Letters 556, 236-244 (2004).
